# Balancing Inbreeding and Outbreeding Risks to Inform Translocations Throughout the Range of an Imperiled Darter

**DOI:** 10.1111/eva.70088

**Published:** 2025-03-23

**Authors:** Brendan N. Reid, Jordan Hofmeier, Harry Crockett, Ryan Fitzpatrick, Ryan Waters, Sarah W. Fitzpatrick

**Affiliations:** ^1^ Department of Ecology and Evolution University of California Santa Cruz Santa Cruz California USA; ^2^ Department of Ecology, Evolution, and Natural Resources Rutgers University New Brunswick New Jersey USA; ^3^ Kansas Department of Wildlife and Parks Pratt Kansas USA; ^4^ Colorado Parks and Wildlife Fort Collins Colorado USA; ^5^ W.K. Kellogg Biological Station Michigan State University Hickory Corners Michigan USA; ^6^ Department of Integrative Biology Michigan State University East Lansing Michigan USA; ^7^ Ecology, Evolution, and Behavior Program Michigan State University East Lansing Michigan USA

**Keywords:** conservation planning, genetic rescue, indels, small populations, streamscape genomics

## Abstract

Restoring connectivity via assisted migration is a useful but currently underused approach for maintaining genetic diversity and preventing extirpations of threatened species. The use of assisted migration as a conservation strategy may be limited by the difficulty of balancing the benefits of reconnecting populations (including reduced inbreeding depression and increased adaptive capacity) with the perceived risk of outbreeding depression, which requires comprehensive knowledge of the landscape of adaptive, neutral, deleterious, and structural variation across a species' range. Using a combination of reduced‐representation and whole‐genome sequencing, we characterized genomic diversity and differentiation for the Arkansas Darter ( *Etheostoma cragini* ) across its range in the Midwestern US. We found strong population structure and large differences in genetic diversity and effective population sizes across drainages. The strength of genetic isolation by river distance differed among drainages, with landscape type surrounding streams and impoundments also contributing to genetic isolation. Despite low effective population sizes in some populations, there was surprisingly little evidence for recent inbreeding (based on the absence of long runs of homozygosity) or for elevated levels of deleterious variation in smaller populations. Considering neutral, adaptive, deleterious, and structural variation allowed us to identify several potential recipient populations that may benefit from translocations and potential donor sites throughout the range. Planning translocation strategies intended for restored connectivity and possible genetic rescue at earlier stages in species decline will likely increase the probability of retaining genetic diversity and population persistence over the long term while minimizing risks associated with translocation.

## 1 Introduction

Extirpation of small, isolated populations is a major driver of biodiversity loss and species extinction (Ceballos et al. 2015). Habitat loss, fragmentation, and climate change lead to smaller and more isolated populations, which are in turn more vulnerable to extirpation due to classic small population problems (Gilpin and Soulé 1986). For example, inbreeding depression risk increases with decreasing population size and rates of immigration (Keller and Waller 2002). Inbreeding plus strong genetic drift can fix deleterious variation in a population, reducing fitness and limiting adaptive potential (Lande 1994). Furthermore, small populations may lack sufficient resiliency to withstand stochastic events such as diseases or climate anomalies (Lande 1993). Whereas translocations have long been used to demographically boost populations (Leberg and Ellsworth 1999; Katzner et al. 2012; Hayes and Banish 2017), a management strategy that is often discussed but rarely implemented is assisted migration into an existing recipient population with the explicit intention of restoring connectivity and, potentially, genetic rescue (Fitzpatrick et al. 2023; Frankham 2015; Ralls et al. 2020). Genetic rescue is an increase in population fitness caused by the immigration of new alleles or gene flow (Bell et al. 2019; Tallmon et al. 2004; Whiteley et al. 2015). However, designing translocation strategies aimed at genetic rescue in conservation practice is typically used only as a last resort and is almost always “genomically” uninformed. That is, rarely are decisions about source and recipient populations informed by rangewide genomic datasets (Fitzpatrick and Funk 2021).

A concern that limits the use of assisted migration for genetic rescue in conservation is the possibility that crossing distinct populations may lead to outbreeding depression (Edmands 2007). Such reductions in fitness can occur due to the disruption of beneficial epistatic effects of co‐adapted genes (Frankham et al. 2017; Whitlock et al. 1995) or through the introduction of genes that are maladapted to local conditions (Aitken and Whitlock 2013). Although there is strong theoretical support for outbreeding depression (Edmands and Timmerman 2003) and examples from wild populations (Houde et al. 2011; Marshall and Spalton 2000; Edmands 1999; Fenster and Galloway 2000; Waser et al. 2000; Whitlock et al. 2013), detrimental effects of outbreeding tend to predictably increase with genetic, geographic, and/or ecological distance as well as time since divergence (Frankham et al. 2017). In spatially structured populations, more physically distant populations likely diverged longer ago, have accumulated more genetic differences, and thus are probably more likely to have differences in interacting genes involved in intrinsic coadaptation. Distance may also cause populations to diverge in genetic regions that are involved in local adaptation, although ecological distance is likely more important than geographic distance in determining the degree of divergence (Leimu and Fischer 2008). Finally, gene flow between populations that occupy starkly different environments is more likely to have a maladaptive effect than between populations in similar environments (Frankham et al. 2011). Frankham et al. (2011) provided guidelines for minimizing outbreeding, which include only crossing populations of the same species with no fixed chromosomal differences that have had gene flow within the past 500 years and have been separated for < 20 generations if there are significant environmental differences.

Outbreeding and inbreeding depression have most often been quantified at the level of individual population crosses in controlled settings (e.g., Edmands 1999). In conservation scenarios, on the other hand, where multiple potential recipient and donor populations exist, identifying the relative risks of outbreeding and inbreeding is considerably more complex and may not be feasible using traditional tools like experimental crosses. However, next‐generation sequencing datasets have expanded the ways in which conservation practitioners can assess factors that would increase outbreeding depression risk. Genomic datasets can be used to infer phylogeographic and phylogenetic history (McCormack et al. 2013) and to estimate divergence histories under multiple complex scenarios (Jackson et al. 2017). Mapping genomic data to increasingly available reference genomes allows for the identification of fixed differences in chromosomal structure among populations (Escaramís et al. 2015). Landscape genomics can be used to identify how genetic diversity is influenced by the environment, revealing barriers to gene flow and patterns of local adaptation across environments that can then be used to guide translocation strategies. Finally, next‐generation sequencing has led to tools for identifying patterns and risks associated with inbreeding, such as characterizing runs of homozygosity for determining a population's inbreeding history (Ceballos et al. 2018). Recent studies have also estimated genetic load dynamics within populations using whole genomes, even without fitness data (i.e., Mathur and DeWoody 2021; Mathur, Tomeček, et al. 2023). Altogether, the wealth of information provided by modern genomic datasets can substantially improve the successful implementation of translocation and genetic rescue strategies, although this potential has not yet been realized in most systems (Fitzpatrick et al. 2023).

Freshwater organisms living in river or stream systems are particularly interesting for studying the dynamics of inbreeding and outbreeding depression. These organisms are naturally dispersal‐limited by the topology of the aquatic networks they inhabit. This network structure often constrains gene flow, limiting the paths and sometimes the direction in which organisms can move (Thomaz et al. 2016). Rivers are also highly vulnerable to fragmentation. Populations that were once continuously distributed in river systems can become fragmented due to local extirpation of intervening (meta)populations or the introduction of barriers to river flow or dispersal through natural means (e.g., oxbows) or anthropogenic means (e.g., dams). Reduced connectivity in fragmented stream systems can lead to reduced overall diversity, increased inbreeding, reduced fitness, and extirpation or extinction (Brauer and Beheregaray 2020; Fagan 2002). Unsurprisingly, nearly 40% of federally threatened or endangered vertebrate species in the US are freshwater fish (Waples et al. 2013). Despite potential risks of outbreeding, assisted migration informed by genomics may represent the best opportunity for restoring gene flow, maintaining adaptive diversity, and avoiding the extinction vortex in fragmented freshwater habitats (Funk et al. 2019; Pavlova et al. 2017).

Our study focuses on the Arkansas Darter (Percidae: *Etheostoma cragini* ; Gilbert 1885), a Great Plains fish species that is threatened throughout its range by anthropogenic impacts on its stream habitats. Arkansas Darters have a disjunct distribution in the greater Arkansas River basin of the central U.S., ranging from eastern Colorado to Arkansas. This species primarily occurs in isolated populations in headwaters and small tributaries to higher order rivers (Labbe and Fausch 2000; Miller 1984). Surveys throughout the Colorado extent of the species range indicate that Arkansas Darter populations are dynamic and exhibit characteristics of a metapopulation with periodic extinction and recolonization in which contiguous streams and rivers serve as dispersal corridors (Loeffler et al. 1982; Loeffler and Krieger 1994). However, factors such as habitat loss, drought, groundwater removal, and impoundments have altered Arkansas Darter connectivity patterns and contributed to population declines throughout the range. Several subpopulations where the species was previously detected have contracted or been extirpated throughout Colorado, southwestern Kansas, and northwestern Oklahoma, where stream flows have been diminished by a combination of climate change and irrigation pressures (Eberle and Stark 2000). Arkansas Darters were designated a candidate species for federal listing from 1991 to 2016 and receive state‐level protection in every state in which they occur (Eberle and Stark 2000; Groce et al. 2012; Hargrave and Johnson 2003; Miller 1984). Ongoing management activities for this species include habitat conservation, hatchery propagation, reintroduction, and re‐establishment of populations, as well as long‐term monitoring. Previous genetic work revealed tiny effective population sizes and found that stream intermittency impeded gene flow among Colorado populations (Fitzpatrick et al. 2014), prompting management discussions about the potential for assisted migration to both restore connectivity and increase genetic variation in small, isolated populations of Arkansas Darters. To date, however, these discussions have lacked a genetically defined framework for understanding population structure across the range of the Arkansas Darter. A previous status assessment for Arkansas Darters (U.S. Fish and Wildlife Service [USFWS] 2016) posited a hierarchical population structure consisting of 15 metapopulations for the species based on nested hydrological unit code (HUC6 and HUC8) designations. While these designations are consistent with expectations for stream‐living organisms, none of these designations were informed or confirmed with genetic information. The goal of this study was to merge landscape genomics approaches with the evaluation of inbreeding and outbreeding risks throughout the species range to define genetic populations and inform management decisions.

We combined whole genome sequence data with a reduced‐representation sequence capture approach (i.e., Rapture, Ali et al. 2016) to achieve both high genomic resolution and large sample sizes throughout the species range. Armed with these datasets, we used a landscape genomic framework to help inform (1) which populations would benefit most from assisted migration and gene flow (i.e., low genetic variation and *N*_e_, high isolation or inbreeding); and (2) identify the best source populations to use for assisted migration (i.e., low neutral and adaptive divergence, minimal structural variation; Table 1). We were also interested in understanding what landscape features cause genetic isolation and affect population size in Arkansas Darters so that these may be targeted for improvement in the future. Our approach is designed to be useful for the conservation and management of this imperiled species, but also to generally serve as a model for using landscape genomics to inform difficult questions in the design and implementation of assisted migration for genetic rescue.

**TABLE 1:** Desirable characteristics of populations potentially receiving and donating individuals for managed gene flow.

Potential gene flow recipient characteristics		Potential gene flow donor characteristics	
Neutral genetic diversity	Low	Neutral genetic diversity	High
Deleterious variation	High	Deleterious variation	Low
Inbreeding	High	Inbreeding	Low
Population size	Low	Divergence relative to recipient	Low
Isolation	High	Structural variants relative to recipient	Low

## 2 Methods

### 2.1 Sampling

Dipnetting and electrofishing were used by Kansas Department of Wildlife and Parks personnel to collect 2374 *E. cragini* individuals at 216 sites throughout Kansas in 2015–2016. Fin clips were taken from adults (> 28 mm) and whole specimens were collected for juveniles (< 28 mm). Samples were stored in 100% ethanol, shipped to Michigan State University (MSU), then stored in a freezer (−20°C) prior to analysis. In addition to the Kansas samples, 60 whole *E. cragini* specimens were collected from six sites in Arkansas by the Arkansas Fish and Game Commission. Tissue samples and DNA from individuals collected by the Colorado Department of Parks and Wildlife were also available from a previous study (Fitzpatrick et al. 2014). Ultimately, we included samples from 14 of the 15 USFWS‐designated metapopulation units (USFWS 2016), as we were unable to obtain samples from the Grand Lake area in Oklahoma. Information for samples retained in the final analysis is provided in Table S1.

### 2.2 DNA Extraction and Genotyping

The wet lab workflow used to extract DNA and obtain genomic data from samples is described in Reid et al. (2021). Briefly, we conducted high‐throughput DNA extractions using a KingFisher Flex DNA extraction system (Thermo Fisher Scientific) to extract DNA from 1635 *E. cragini* samples from Kansas, 60 *E. cragini* samples from Arkansas, and 20 *E. cragini* samples from Colorado. DNA extracted for this study from *E. cragini* covered a total of 232 collection sites (*n* = 2–10 per site; Table S1). To this dataset, we added 140 previously extracted DNA samples from 11 sites in Colorado, including samples from hatcheries and reintroduced populations that were not used in this study. DNA yields were assessed using a PicoGreen assay (Thermo Fisher Scientific). We used two different genotyping approaches: (1) Rapture (Ali et al. 2016), which combines RADseq and sequence capture to efficiently genotype a set of target loci from a large number of individuals; and (2) moderate‐coverage whole‐genome sequencing (WGS, with a target coverage of ≥ 5×), which we applied to a representative subset of individuals to obtain detailed genome‐scale datasets. For Rapture genotyping, we used the BestRAD protocol along with a sequence capture step (Ali et al. 2016) using baits previously tested on multiple darter species (Reid et al. 2021) to conduct reduced‐representation library preparation using NEBNext Ultra DNA Library Prep kits (New England Biosciences) for 1855 *E. cragini* individuals. Five lanes of sequencing were used in total for Rapture genotyping. For WGS genotyping, we submitted isolated DNA from 24 *E. cragini* samples from 10 river drainages (5 individuals from the Ninnescah River; 3 individuals each from the Chikaskia, Cimarron, and Lower Arkansas rivers; 2 individuals each from the Salt Fork Arkansas River and Walnut Creek; and 1 individual each from Big Sandy Creek, Spring River, Upper Arkansas River, and below the confluence of the Arkansas and Ninnescah rivers; Table S2) for whole‐genome resequencing at the MSU Core Genomics center over two sequencing lanes. All sequencing was performed on the Illumina HiSeq 4000 platform. All sequences were mapped to the chromosome‐level Arkansas Darter reference genome (CSU_Ecrag_1.0; length = 643.1 Mb, N50 = 27.59 Mb; Reid et al. 2021) generated from a Colorado hatchery individual following quality control as described in Reid et al. (2021).

### 2.3 SNP Calling and Filtering

For the Rapture dataset, we used ANGSD (Korneliussen et al. 2014) to calculate genotype likelihoods for all SNPs, using a *p* value of 1e‐6, SNPs genotyped in ≥ 80% of individuals, and with a minimum MAF of 0.05. 6770 SNPs passed this initial filtering threshold. We then applied several filters to remove SNPs that were closely linked. First, we removed any SNPs that were within 2 kb of one another (i.e., SNPs on the same Rapture loci). We used NGSLD to calculate linkage disequilibrium based on genotype likelihoods for the remaining SNPs. We pruned any SNPs on the same chromosome with a weight > 0.5, leaving a final linkage‐pruned data set of 815 SNPs. After initial population structure analyses (Reid et al. 2021) we identified 18 individuals (approximately 1% of the total number genotyped) that clustered with a drainage other than the drainage in which they were sampled—due to the low likelihood of natural dispersal among these drainages, we assumed these may have been due to sample mislabeling or lab error, and we removed them from further analyses. We also removed any individuals with < 80% complete data and a small number of hatchery‐raised fish, leaving a final Rapture dataset of 1643 individuals.

For the WGS dataset, we used FastQC (http://www.bioinformatics.babraham.ac.uk/projects/fastqc/) to assess sequencing quality across individuals. We used BWA v.0.7.17‐r1188 (Li and Durbin 2009) to align sequences to the *E. cragini* genome. We used samtools v.1.9 (Li et al. 2009) to filter out low‐quality sequences and improperly paired reads, remove duplicates, and compute average coverage over the whole genome and over all covered sites. For WGS analyses that required called SNPs, we used ANGSD to call SNPs for sites with a *p* value of < 1 × 10^−6^ of containing a SNP in the dataset, and we used a posterior probability cutoff of 0.99 and a minimum depth of 10 (given a mean depth of approximately 11× across WGS samples; Reid et al. 2021) for calling each SNP for each individual.

### 2.4 Rapture: Population Structure and Environmental Associations

We used three parallel methods (snmf, PCA, and differentiation measured by *F*_ST_) to define population structure across the range of the Arkansas darter. To estimate broad‐scale population structure, we used the snmf algorithm (Frichot et al. 2014) implemented in the R package LEA v.3.18.0 (Frichot and François 2015). We assessed structure over a range of values for *K*, corresponding to the assumed number of populations contributing to the ancestry of the sampled individuals. We set a minimum bound of 2 (the lowest meaningful value for *K*) and a maximum of 20, corresponding to a greater number of ancestral populations than the 15 previously identified USFWS metapopulations based on hydrological units. We ran a total of 10 replicate runs of snmf per *K* value with a maximum of 200 iterations per run. We examined cross‐entropy for each value of *K* and identified values of *K* for which the mean cross‐entropy across runs did not decrease with increasing *K* (conservative estimate) and the lowest overall value of *K* (liberal estimate). We performed a principal components analysis (PCA) using the dudi.pca function in ade4. We retained 20 PC axes and visualized PCA results over all of these axes. We also calculated Watterson's *F*_ST_ for each pair of metapopulations (excluding some sites in the Lower Arkansas River that displayed mixed ancestry; see Section 3) using the R package hierfstat (Goudet 2005). We estimated confidence intervals for *F*_ST_ values by bootstrapping over loci 100 times.

To identify SNPs that could be under selection and exclude these SNPs from further analyses, we used LFMM (Frichot and François 2015) to test for associations between SNPs and environmental factors while accounting for population structure. We evaluated associations for three climatic factors that exhibit strong and spatially divergent gradients across the study area (Figure S1a): annual mean temperature (strong cold‐to‐warm gradient from north to south), annual precipitation (strong wet‐to‐dry gradient from east to west), and temperature seasonality (highest seasonality at the eastern and western extremes of the range). We used the more conservative estimate of population structure (*K* = 8) to increase the power to detect SNPs with environmental associations (Forester et al. 2018), and we imputed missing data using this value of *K*. We ran 5 iterations for each climate variable. We assessed significance using a Bonferroni‐Holm correction for multiple comparisons.

### 2.5 Landscape Associations With Genetic Diversity and Distance

We identified a priori a set of landscape variables that we believed could affect within‐site genetic diversity and pairwise genetic differentiation among populations. Different stream metapopulations may have different histories of colonization and effective population size trajectories, and as such, the metapopulation of origin could affect both diversity and divergence. Pairwise stream distance among populations has been shown in many freshwater systems to be correlated with genetic distance. If gene flow is unidirectional, upstream populations should also have lower diversity than downstream populations (Thomaz et al. 2016). Stream intermittency has been shown to affect genetic isolation among Arkansas darter populations in Colorado (Fitzpatrick et al. 2014). Other alterations to stream flow, including dams and impoundments, will also likely affect gene flow among sites. Finally, although the effects of upland habitats have rarely been studied in streamscape genetics, the surrounding landscape could affect connectivity and population size as well. Agricultural landscapes could have altered patterns of runoff or water diversion, and developed landscapes with increased amounts of impervious surface could also alter streamflow.

We calculated *H*_s_ (expected site‐level heterozygosity) for each site and linearized Nei's *F*_ST_ for each pair of sites using the R package hierfstat (Goudet 2005). We used the National Hydrography Dataset (NHD; U.S. Geological Survey 2023) to define river networks, and we used the R package riverdist (Tyers 2017) to clean networks and to identify stream paths between each pair of sites within each genetically defined metapopulation. We calculated upstream distance for a site as the distance between that site and the furthest downstream site within the same genetically defined metapopulation, and we used the NHD to classify each site as either intermittent or perennial. To identify flow obstructions, we used a shapefile of Kansas dams to find all headwater sites that were upstream of a dam, and we added a binary factor to identify dammed sites. Cheney Reservoir Dam creates a large reservoir into which several study streams flow, and we identified these streams with another binary factor. We characterized the landscape within a 500 m buffer around either each site or each pairwise stream path using the National Landcover Database (U.S. Geological Survey 2014) by counting the number of pixels classified into four terrestrial land cover categories (developed/impervious, grassland, cropland, and forest) and dividing by the total number of pixels for each buffer.

To identify associations between genetic characteristics and stream/landscape, we used linear regressions. We excluded genetically defined metapopulations with four or fewer total sites (corresponding to six or fewer pairwise *F*_ST_ values) from this analysis, and we excluded one site in the Big Sandy Creek that had a very high *F*_ST_ compared to all other sites in its genetically defined metapopulation. We first identified a global model containing all variables. We used the “dredge” function in the R package MuMin to fit reduced models and rank these by AIC. To assess the potential for multicollinearity, we calculated correlations among continuous predictor variables for each model in R. Given the nonindependence of pairwise distance data structures (Wang 2013) when using linearized *F*_ST_ as the response variable, we tested for significance using a permutation approach. Within each genetically defined metapopulation, we randomly permuted the linearized *F*_ST_ values and fit the best model again. We evaluated *R* ^2^ for each fitting of the models with randomized data, and we compared the distribution of randomized *R* ^2^ values to the observed *R* ^2^ value.

### 2.6 Effective Population Sizes

We used the program NeEstimatorv2 (Do et al. 2014) to infer effective population sizes (*N*_e_) using the linkage disequilibrium (LD) method. Linkage disequilibrium estimates of effective population size best reflect the local effective size when the unit of analysis is a single panmictic population and migration rates into this population are low (< 5%–10% per year; Waples 2010; Waples and England 2011). When populations are genetically structured, LD‐based estimates of *N*_e_ will be downwardly biased due to Wahlund effects. Small sample sizes relative to census population size can also cause downward biases in estimates of *N*_e_ (Waples 2006), although these biases can be and are corrected for in NeEstimator (Waples and Do 2008; Do et al. 2014). To account for these potential biases, we grouped samples at the scale of HUC10 watersheds, which are nested within HUC8 watersheds and represent the units originally defined as “populations” respectively, in the Arkansas darter SSA (USFWS 2016). Within these units, we assessed all pairwise *F*_ST_ values (see above) and iteratively removed populations with the highest average pairwise *F*_ST_s until the average *F*_ST_ was < 0.02 using a custom R script. After pruning populations with high *F*_ST_s, we removed any units with 30 or fewer individuals. In all cases, we used a minor allele frequency cutoff of 0.05, and we calculated 95% confidence intervals for *N*_e_ using the jackknife method. As the HUC10 units used here are open to migration from other populations in the same stream system, the *N*_e_ estimates likely represent metapopulation‐level *N*_e_ rather than local *N*_e_ (Waples 2010; Waples and England 2011).

### 2.7 Whole‐Genome Diversity and Runs of Homozygosity

Long runs of homozygosity (ROH) due to large sections of the genome that are identical by descent are considered indicative of recent inbreeding (Ceballos et al. 2018; Curik et al. 2014). To identify ROH from our medium‐coverage WGS data, we used the ROH detection function in plink v.1.9 (Chang et al. 2015), which classifies sections of the genome as ROH or non‐ROH based on the number of heterozygotes within a sliding window. Heterozygotes in an ROH could represent sequencing errors or germline mutations. We used a window size of 100, a minimum ROH length of 100 kb, and a maximum number of heterozygotes per window of 1 to identify the amount of the genome in ROH according to plink (ROH_P_). We also calculated heterozygosity for each individual and compared this to the total length of the genome in ROH.

Plink does not account for ROH expected due to low overall genetic diversity (i.e., ROH that are identical by state [IBS] but not due to recent IBD), which could be a confounding factor given the large rangewide variation in diversity observed for *E. cragini* . To examine this, we used the program ROHan (Renaud et al. 2019). ROHan co‐estimates overall genetic diversity (theta), the proportion of the genome in ROH due to recent IBD, and the length of ROH. We set the minimum ROH length to 100 kb. We compared estimated theta to the estimated proportion of the genome in ROH according to ROHan (ROH_R_) for each individual.

### 2.8 Structural Variation

Chromosomal differences are considered to be potentially detrimental to the effectiveness of assisted migration and genetic rescue as they increase the probability of outbreeding depression (Frankham et al. 2011). We used the program Manta v.1.6 (Chen et al. 2016) with default settings to detect the number of structural variants relative to the Arkansas Darter reference genome for each individual with WGS data. From the called diploid structural variants identified by Manta for each individual, we identified the number of insertions and deletions (indels) and the length of each of these variants. We also counted the number of potential inversions or translocations (designated by breakend variants) and tandem duplications identified for each individual. As the reference genome was constructed from a Colorado individual and Colorado populations are potential high‐priority targets for assisted migration, we compared the number of indels detected to the divergence between each drainage and the Colorado populations using linear regression, using genetically defined metapopulation‐level *F*_ST_ calculated from Rapture loci and divergence time relative to Colorado populations (estimated in Reid et al. 2021) as two alternate metrics of divergence. For a single individual from the Arkansas/Ninnescah confluence genetically defined metapopulation, which appears to represent admixture between the Arkansas and Ninnescah River genetically defined metapopulations, we averaged the divergence times calculated in Reid et al. (2021) for these two ancestral populations as a divergence time metric.

### 2.9 Evaluating Genome‐Wide Functional Variants Across the Range

To identify and categorize SNPs with potential functional effects for the purpose of evaluating genetic load, we applied the program SnpEff (Cingolani et al. 2012) to our WGS dataset. We first created a SnpEff database using the sequence data (.fa) and annotation file (.gtf) from the Arkansas Darter reference genome. We then used SnpEff to annotate all variants. We divided variants into four categories: high‐effect (introducing stop codons or frameshift mutations into a coding sequence), moderate‐effect (nonsynonymous substitutions in a single amino acid or in‐frame indels in a coding sequence), low‐effect (synonymous changes in a coding sequence), and noncoding variants. For each individual, we calculated the proportion of deleterious variants as the total number of variants in the first three classes divided by the total number of noncoding variant SNPs genotyped for that individual. We used a linear regression to assess whether the proportion of deleterious variants was associated with the heterozygosity of noncoding variants reported by SnpEff. No association between the total number of variants genotyped and the proportion of variants in each category would indicate that for a given SNP, all individuals are equally likely to carry a similar number of alleles of a given effect. A negative association would indicate a higher proportion of SNPs associated with deleterious effects in individuals with less diversity, while a positive association would indicate that deleterious SNPs represent a smaller portion of total SNPs in individuals with lower diversity (which would be expected under purging of inbreeding load; e.g. Grossen et al. 2020; Robinson et al. 2018, 2022).

## 3 Results

### 3.1 WGS and Rapture Dataset Descriptions

The 815 SNPs in the Rapture dataset had 5.3% missing data, with the amount of missing data roughly similar among genetically defined metapopulations (Figure S2), and were sequenced with depth > 50× across most individuals (Reid et al. 2021). Overall heterozygosity across all complete genotypes was 21.3%. For the WGS dataset, the mean per‐site depth across all individuals was 10.2 (individual range = 8.05–13.81), and for called genotypes, the mean amount of missing data across all individuals was 59.6% (range = 28.5%–80.4%).

### 3.2 Strong Drainage‐Scale Population Structure and Little Evidence for Selection

PCA analysis showed spatial clustering among individuals on the first 14 axes (Figure S3a–g), after which there was little observable clustering (Figure S3h–j). The first PC axis differentiated individuals in the Arkansas River drainages from individuals in other drainages, with individuals in Slate Creek and below the Arkansas/Ninnescah confluence intermediate (Figure S3a). The second axis differentiated most of the other drainages (although the Medicine Lodge and Ninnescah drainages grouped together; Figure S3a). PC 3 differentiated the Illinois and Spring River populations, and PCs 4 and 5 differentiated the Medicine Lodge drainage from the Ninnescah drainage (Figure S3b,c). The Colorado sites (Upper Arkansas, Middle Arkansas, and Big Sandy/Rush Creek drainages) were differentiated from Lower Arkansas sites on PC 6 (Figure S3c). Additional PC axes differentiated the Salt Fork Arkansas River (Figure S3d), Walnut Creek, Spring River, Slate Creek (Figure S3e,f), Upper Arkansas River, and Big Sandy/Rush Creek drainages (Figure S3g).

**FIGURE 1:**
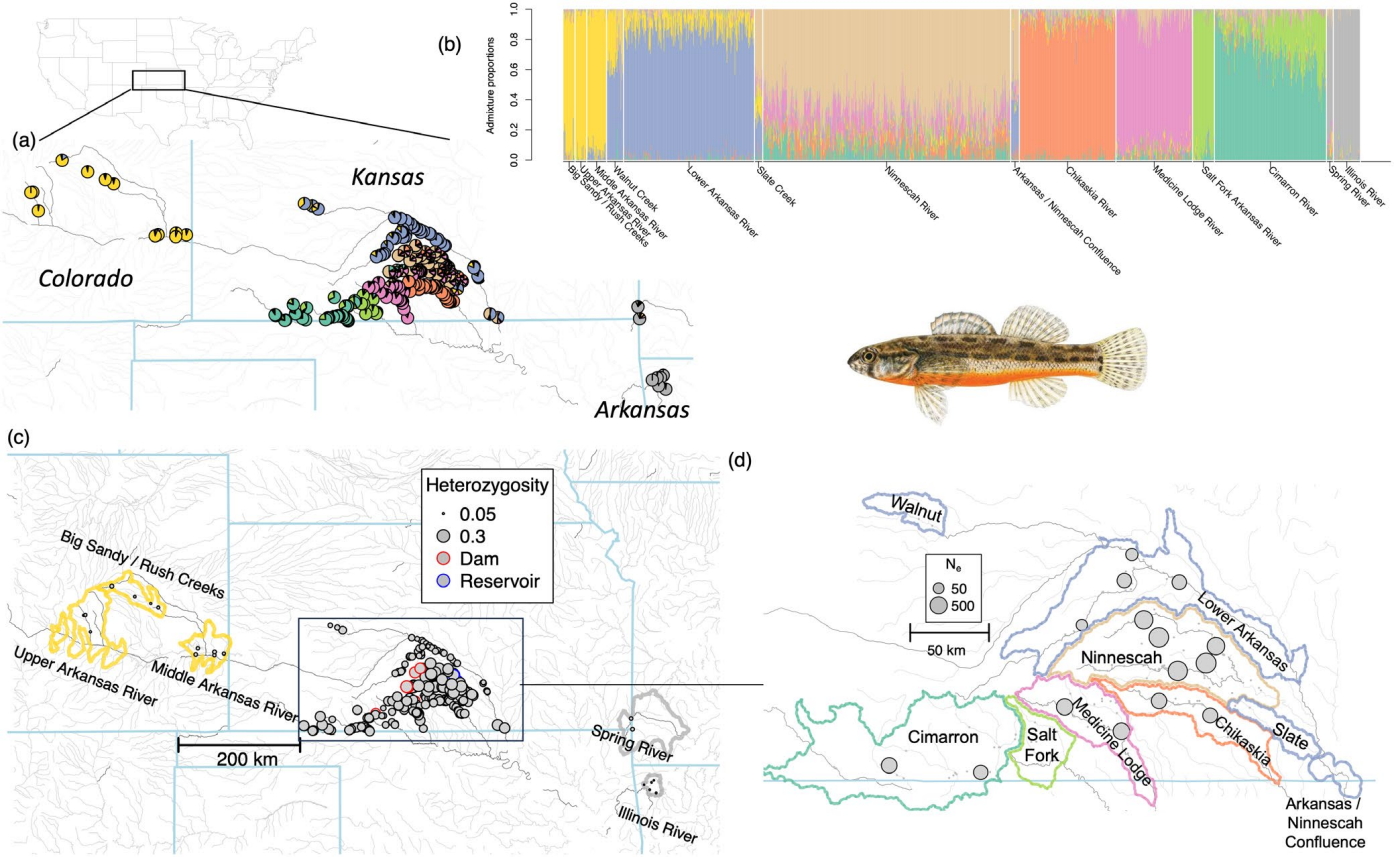
Structure barplot and maps of admixture proportions, heterozygosity, and effective population size for each site based on Rapture data. (a) Pie charts of combined admixture proportions for all individuals at each site sampled, with colors corresponding to colors used in (b). Location of study are within US is shown above. (b) Barplots showing admixture proportions for each individual genotyped. Individual are grouped by genetically defined metapopulation of origin and ordered by decreasing latitude within each metapopulation. (c) Mean site‐level heterozygosity, with circle size proportional to heterozygosity. Sites above dams are shown with red borders, while sites adjacent to the Cheney Reservoir are shown with blue borders. (d) Estimated effective population size for HUC10 drainages, with circle size proportional to log‐transformed *N*_e_.

For the snmf population structure analysis, cross‐entropy tended to decrease with increasing values of *K*. The lowest value of *K* was at *K* = 16, although cross‐entropy only slightly decreased and reached an asymptote after *K* = 8 (Figure S4). At *K* = 8, sites generally displayed inferred ancestry corresponding to USFWS metapopulation structure (Figure 1a,b). Population structure and PCA axes largely agreed on the spatial structuring of genetic diversity for the Arkansas darter. Colorado sites (Upper Arkansas, Middle Arkansas, and Big Sandy/Rush Creek drainages) derived most of their ancestry from the same inferred ancestral population, as did the North and South Fork Ninnescah sites. Most Lower Arkansas and Rattlesnake Creek sites were primarily derived from the same ancestral population; however, Lower Arkansas sites in the Slate Creek assigned with the Ninnescah River, and Lower Arkansas sites below the Arkansas–Ninnescah confluence displayed mixed ancestry. Cimarron River individuals showed mixed ancestry with Salt Fork Arkansas River individuals. Walnut Creek sites exhibited primarily Lower Arkansas ancestry but some evidence of admixture with Colorado.

Using the USFWS metapopulation designations, pairwise *F*_ST_ values were low (< 0.03) between metapopulations with close hydrological connections (Lower Arkansas/Rattlesnake Creek and North/South Fork Ninnescah) ranging to > 0.8 between the spatially distant Illinois River and Colorado populations (Figure S5a). After re‐grouping units to reflect genetically defined populations, the lowest pairwise *F*_ST_ was 0.08 between the Medicine Lodge and Ninnescah drainages (Figure S5b). As reflected above by high levels of population structure, many genetically defined metapopulations showed exceptionally high (> 0.5) levels of pairwise genetic differentiation, indicating little recent connectivity among drainages at this scale, with the highest average divergences observed between the Illinois and Spring River metapopulations and the other metapopulations (Table S3a). No confidence intervals for pairwise *F*_ST_ values among genetically defined metapopulations overlapped zero (Table S3b).

LFMM indicated generally lower *p* values for associations between SNPs and precipitation compared to seasonality or mean temperature (Figure S1b). However, after Bonferroni‐Holm correction, only two SNPs had significant associations with precipitation. Neither of the other variables had *p* values crossing the significance threshold. We removed the two SNPs demonstrating an association with precipitation from further analysis of genetic differentiation and effective population size.

### 3.3 Variable Effective Population Sizes Across the Range

We identified 15 HUC10 watersheds from five genetically defined metapopulations in Kansas (Chikaskia, Cimarron, Lower Arkansas, Medicine Lodge, and Ninnescah) with sufficient sample sizes after pruning for *N*_e_ analyses. Effective population sizes estimated from HUC10 watersheds ranged from 58.1 in the Lower Arkansas River metapopulation to over 1000 in the South Fork of the Ninnescah River (Table 2). Effective size estimates were generally similar across HUC10s within metapopulations. All point estimates from the Lower Arkansas River metapopulation, for example, were < 200, while all estimates from the Ninnescah River were > 500. Effective size estimates for HUC10 watersheds within the Cimarron River metapopulation (181.6–280), Chikaskia River metapopulation (220.5–261), and Medicine Lodge River metapopulation (373.5–397.8) were intermediate. While point estimates were generally fairly consistent among HUC10s within genetically defined metapopulations, confidence intervals were generally large, and upper confidence limits for several Ninnescah River HUC10s were infinite.

**TABLE 2:** Effective population size estimates for HUC10 watersheds within five genetically defined metapopulations.

Metapopulation	HUC10	*N*_e_	95% CI	*n*
Lower Arkansas	Antelope Creek	84.5	59–139.1	45
Lower Arkansas	Peace Creek	192.3	113.5–544.5	48
Lower Arkansas	Salt Creek	185.9	122.7–357.1	63
Lower Arkansas	Wild Horse Creek	58.1	41.5–91.7	33
Cimarron	Day Creek	181.6	92.9–1122.2	60
Cimarron	Lower Crooked Creek	280.8	194.4–489.3	62
Chikaskia	Sand Creek	261	129.8–4890.2	42
Chikaskia	Spring Creek	220.5	164.3–327.7	62
Medicine Lodge	Lower Medicine Lodge	397.8	286.2–637.2	87
Medicine Lodge	Upper Medicine Lodge	373.5	206–1719.3	32
Ninnescah	North Fork Outlet	563.6	242.8–Infinite	45
Ninnescah	Silver Creek	1151.2	382–Infinite	36
Ninnescah	South Fork Headwaters	717.1	428.6–2057.3	78
Ninnescah	South Fork Outlet	1034.2	772.5–1546	156
Ninnescah	Smoots Creek	1290.9	597–Infinite	53

### 3.4 Associations Between Diversity, Divergence, and Landscape

Heterozygosity for Rapture loci varied drastically among genetically defined metapopulations (Figure 1c; Table S1) and was lowest in the Eastern genetically defined metapopulations (Illinois River = 0.038; Spring River = 0.089), Colorado metapopulations (Upper Arkansas = 0.06; Big Sandy/Rush Creeks = 0.07; Middle Arkansas = 0.082), Walnut Creek (0.14), Salt Fork (0.145), and Lower Arkansas (Lower Arkansas = 0.149). Sites in the Ninnescah River (heterozygosity = 0.26) and Medicine Lodge (0.219) had the highest heterozygosity. The best linear model for predicting variation in heterozygosity contained genetically defined metapopulation, upstream distance, and adjacency to the Cheney Reservoir as explanatory variables. Model selection results for heterozygosity showed a sharp contrast between models containing metapopulation as a categorical factor versus models that did not contain metapopulation (Table S4). After accounting for the strong effect of metapopulation on heterozygosity, upstream distances had a positive effect on heterozygosity (the opposite of the expected effect), and this effect was highly statistically significant in the top model (*p* < 0.001) and included in all models with ∆AIC < 10. Populations adjacent to the Cheney Reservoir tended to show lower heterozygosity than expected, although this effect was only marginally significant in the top model (*p* = 0.082) and an effect of the reservoir was not included in all highly ranked models. Although land cover and dams were included in some highly ranked models, they did not explain a significant proportion of variation in heterozygosity. The absolute value of most pairwise correlations among continuous predictor variables in the model for heterozygosity was < 0.4, the exception being proportion (grassland) and proportion (crop) which were negatively correlated (Pearson correlation = −0.648).

**FIGURE 2:**
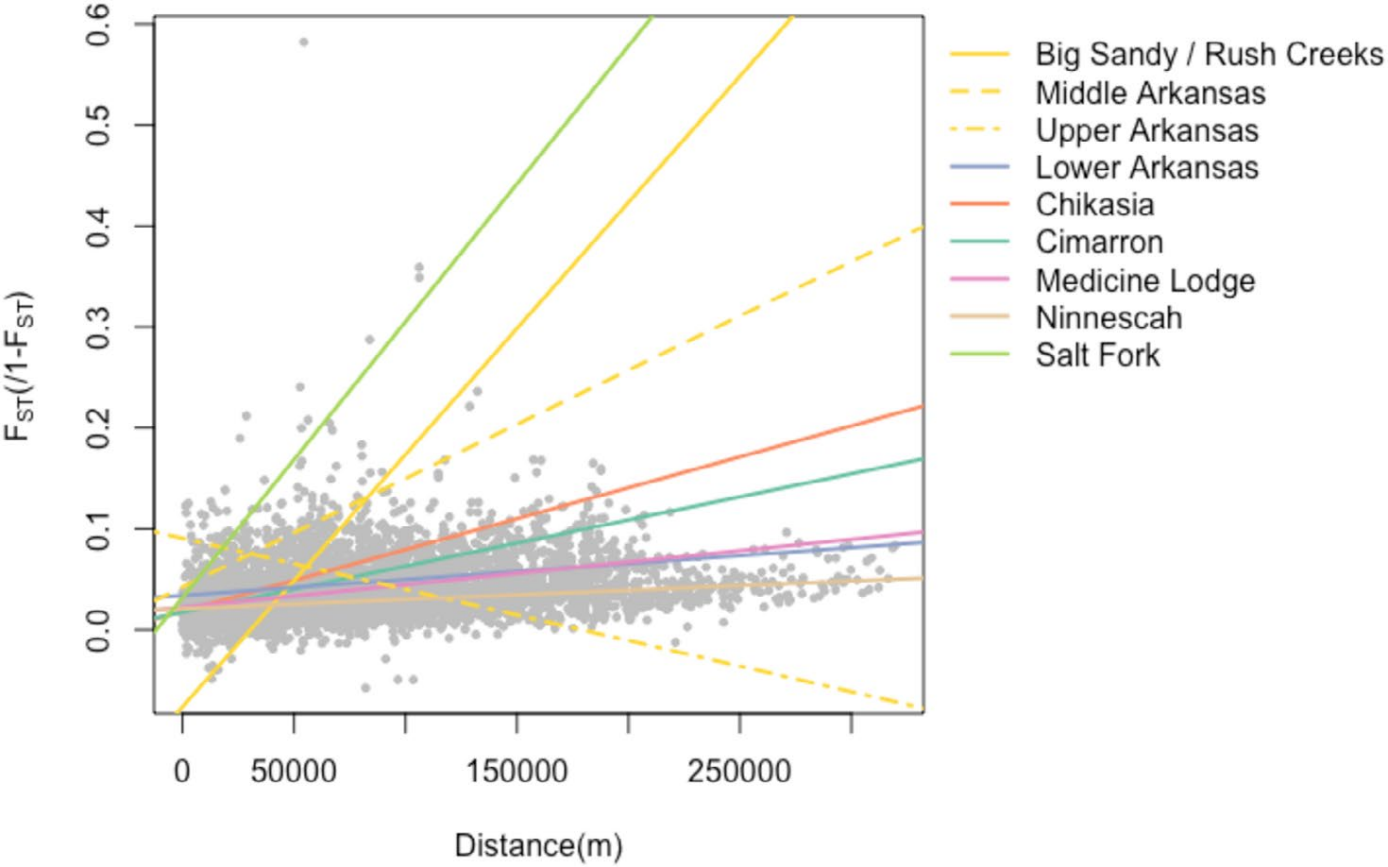
Isolation‐by‐distance within genetically defined metapopulations. Pairwise linearized genetic divergence values (*F*_ST_/1 − *F*_ST_) are shown for each pair of sites within each metapopulation as small gray circles. Fitted relationships are shown as colored lines, with line colors corresponding to metapopulation.

Genetic differentiation among sites within genetically defined metapopulations tended to be low to moderate, with 90% of pairwise *F*_ST_s between 0.002 and 0.093 (median *F*_ST_ = 0.0285); although a small number of pairwise divergences were substantially higher (maximum within‐metapopulation *F*_ST_ = 0.582; Figure 2). In contrast to heterozygosity, model selection results for pairwise divergence indicated a small set of well‐supported models, with only four models with ∆AIC < 2 and all other models with ∆AIC > 8 (Table S5). In all top models, there was a strong distance × metapopulation interaction (Figure 2, Table S5), with different intercepts and slopes for the isolation‐by‐distance relationship across genetically defined metapopulations. All top models with ∆AIC < 2 also contained significant positive associations between differentiation and the proportion of intermittent stream between sites and the proportion of cropland adjacent to the stream, as well as a significant negative relationship between the proportion of developed land and divergence. Although the proportion of grassland and forest cover adjacent to streams was included in some top models, the effects of these land cover variables were not significant. Effects of obstructions to water flow (the Cheney Reservoir as well as smaller dams) were also included in all top models. Sites upstream of dams had higher pairwise divergence with other sites in the same genetically defined metapopulation than otherwise expected. Sites adjacent to the Cheney Reservoir also showed evidence of isolation, with many sites in tributaries to the reservoir displaying much higher pairwise *F*_ST_ values than other pairs of sites in the same drainage and no relation between distance and *F*_ST_ for these sites, suggesting the reservoir is acting as a strong barrier to connectivity (Figure S6). For the divergence model, the absolute values of most pairwise correlations among continuous predictor variables were < 0.4, with the exception being the proportion of grassland and the proportion of forest between sites, which were negatively correlated (Pearson correlation = −0.538).

The best landscape model had an *R* ^2^ of 0.3586. Fitting the same model to permuted datasets, where *F*_ST_ values were shuffled within genetically defined metapopulations, yielded *R* ^2^s ranging from 0.123 to 0.15 (mean = 0.128; Figure S7). This result illustrates that while metapopulation identity explains a substantial amount of variation in pairwise *F*_ST_ on its own, pairwise differences in river distance and landscape variables accounted for more than half of the variance explained in the full model.

### 3.5 Little Evidence for Runs of Homozygosity

**FIGURE 3:**
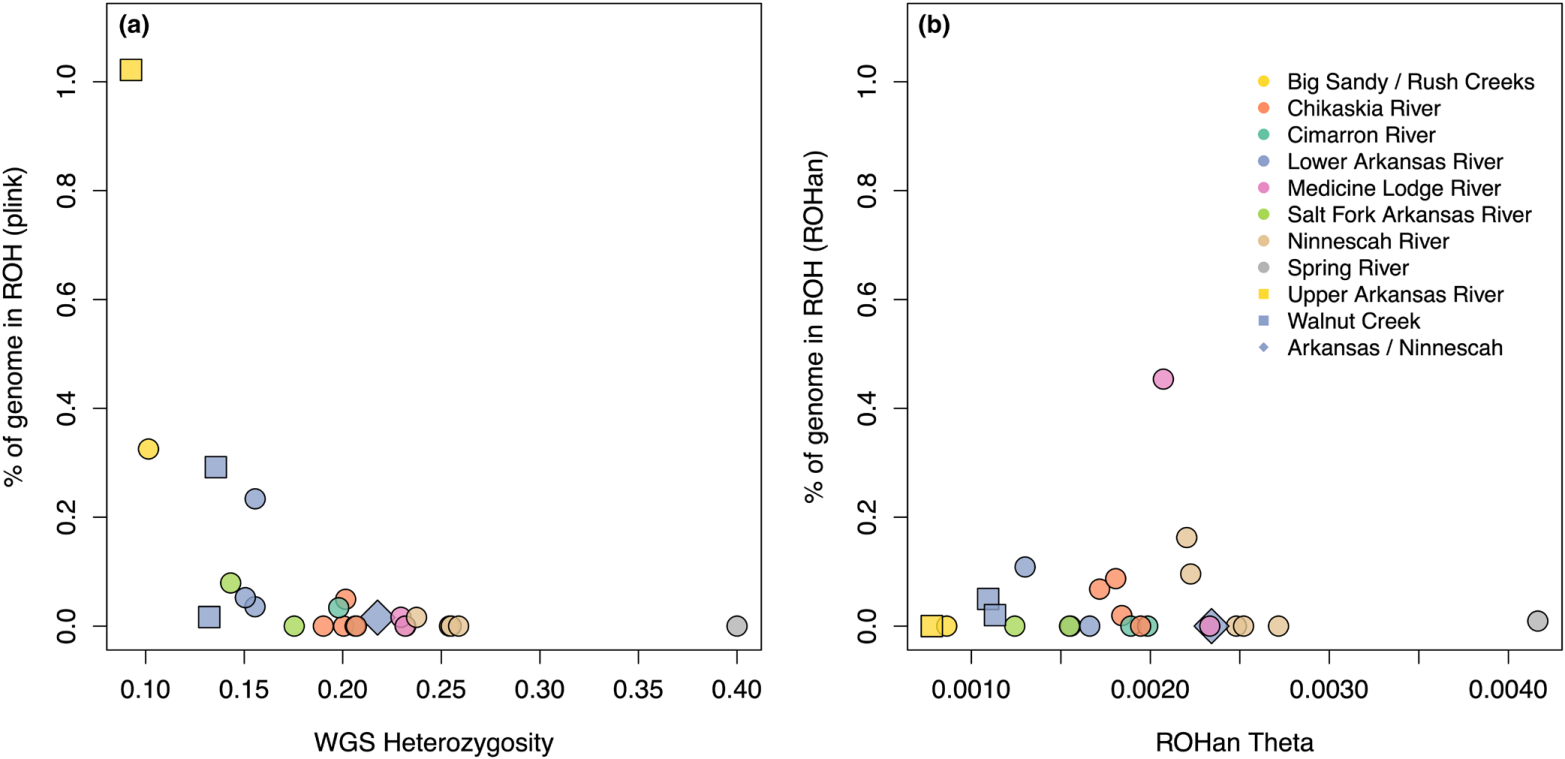
Runs of homozygosity (ROH) plotted against individual genetic diversity calculated using two methods. (a) Percent of genome in ROH estimated by plink versus whole‐genome heterozygosity calculated in vcftools. (b) Percent of genome in ROH versus individual theta estimated by ROHan.

Genome‐wide heterozygosity followed a similar geographic pattern compared to Rapture‐estimated heterozygosity across the range, and the two values were tightly correlated for all genetically defined metapopulations except Spring River, which exhibited high heterozygosity estimated by WGS but low heterozygosity estimated by Rapture (Figure S8). The proportion of the genome in ROH_P_ was extremely low (< 0.1%) in most individuals (Figure 3a). The proportion was somewhat higher in four of the 24 individuals, although the total amount of the genome in ROH_P_ was still low (between 0.1% and 1%) suggesting no recent inbreeding between close relatives in the subset of individuals with their whole genomes sequenced. The four individuals with > 0.1% of the genome in ROH_P_ all had relatively low heterozygosity (~0.1 to 0.15) and were from either the Upper Arkansas or tributaries of the Lower Arkansas.

Theta values inferred by ROHan were highly correlated with genome‐wide heterozygosity (*r* = 0.99; Figure 3b). Similar to ROH_P_, the proportion of the genome in ROH_R_ was also generally low (< 0.5% across all individuals). However, ROH_R_ did not show the same association with diversity as ROH_P_, and Colorado individuals did not show evidence for elevated ROH_R_.

### 3.6 Increasing Structural Differences With Divergence

**FIGURE 4:**
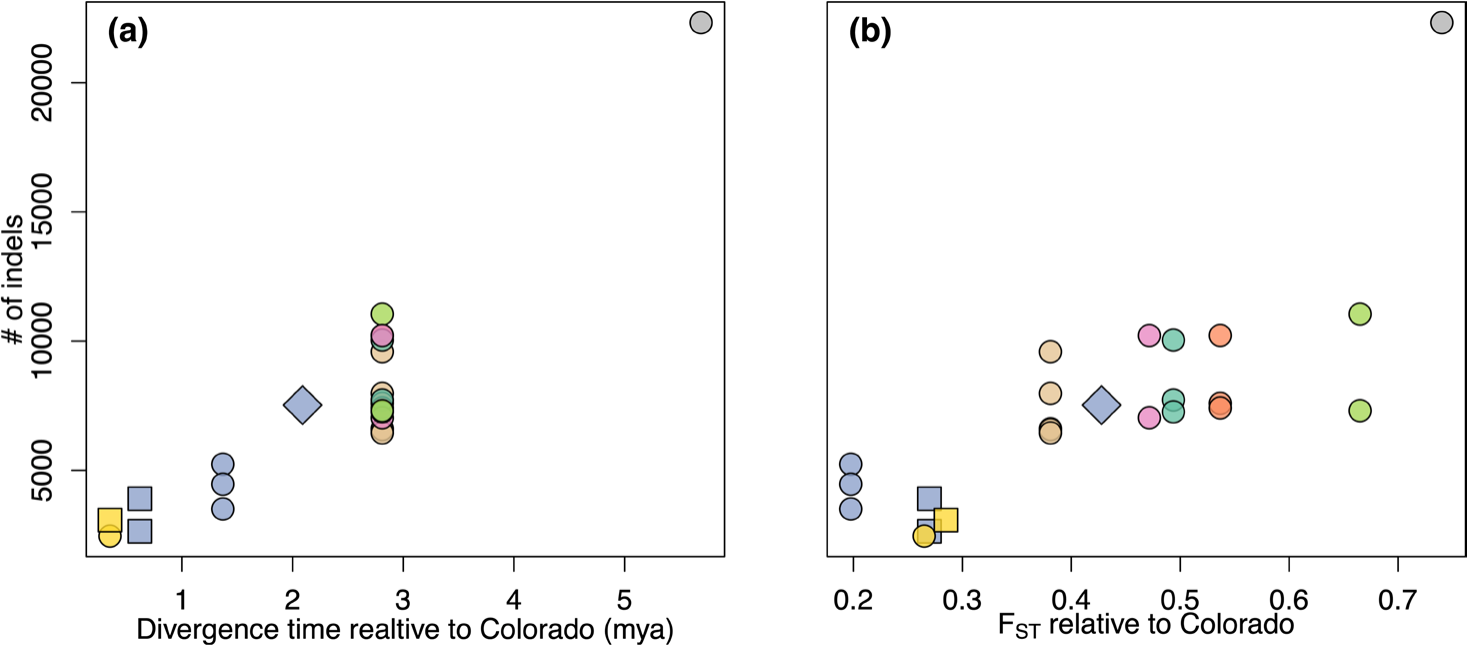
Number of insertions and deletions (indels) relative to the Colorado reference genome plotted against divergence relative to the Colorado individual used to assemble the *E. cragini* reference genome. (a) Number of indels versus divergence time as estimated in Reid et al. (2021). (b) Number of indels versus genetic divergence (*F*_ST_) from the reference individual.

Indels were the most commonly detected structural variant. The number of indels detected in each genetically defined metapopulation relative to the Colorado reference genome increased with increasing genetic distance from Colorado, with fairly small numbers of indels detected for the Arkansas River and its tributaries (Big Sandy/Rush Creek = 2459; Upper Arkansas = 3074; Lower Arkansas = 3506–5238; Walnut = 2641–3902; Figure 4). In other drainages, the number of indels relative to the Colorado reference genome ranged from 6449 in the North Fork of the Ninnescah River to 22,325 in the Spring River. The number of indels detected in each drainage was significantly related to pairwise *F*_ST_ between each drainage and Colorado and to divergence time relative to Colorado (*p* < 1 × 10^−5^ for both). Insertions and deletions tended to be small, and the median size among all insertions and deletions was 95 bp (range = 50–975 bp). Insertions were unimodally distributed, while deletions had a secondary peak around 250 bp, and the distribution of indel sizes was similar among genetically defined metapopulations (Figure S9). Smaller numbers of breakend and duplication variants were detected per individual (up to 500), but these variant types displayed similarly significant positive relationships with divergence (inversion *p* values < 0.001; duplication *p* values < 0.05; Figure S10).

### 3.7 Deleterious Variants Correlated With Diversity

**FIGURE 5:**
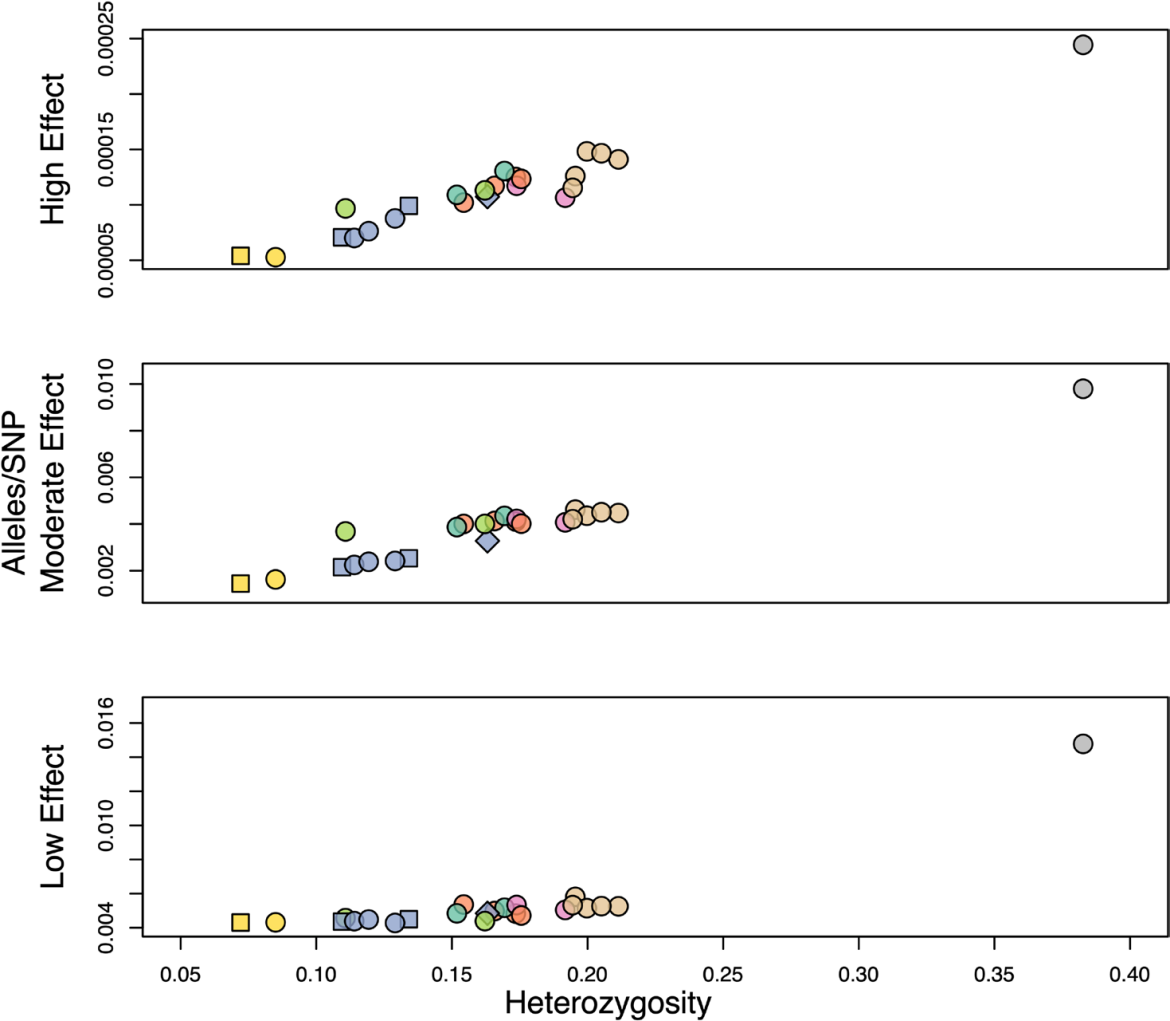
Genome‐wide prevalence of polymorphisms with putative functional effects in each individual for which WGS was performed. The *Y*‐axis shows for each individual the ratio of the summed number of alleles with a given functional annotation (low‐effect, moderate‐effect, or high‐effect) to the total number of SNPs genotyped for that individual. The color/symbol scheme for genetically defined metapopulations matches Figure 4.

The vast majority (~97.14%) of the 2,147,576 variants were annotated as “modifiers” with no putative impact. Low‐effect alleles were slightly more common overall (1.671%) than moderate‐effect alleles (1.163%), and high‐effect alleles were the rarest (< 0.026%) of all. For each category of functional variant detected by SnpEff, there was a statistically significant positive relationship between the proportion of potentially deleterious alleles and individual heterozygosity (Figure 5) consistent with the purging of deleterious variants.

## 4 Discussion

For species facing changing landscapes and multiple spatially heterogeneous threats, rangewide assessment of connectivity and genetic diversity is a key step in the planning and deployment of conservation measures. Populations that are small, lacking genetic diversity, and/or genetically isolated are at the highest risk of extinction, and these populations can be prioritized for measures aimed at increasing population size and connectivity, including assisted migration. Using a combination of rangewide reduced‐representation sequencing and whole‐genome sequencing of a representative subset of populations, we present a thorough assessment of the genetic threats faced by the Arkansas Darter that could serve as a model for other species. We provide recommendations based on this assessment below.

### 4.1 Metapopulation‐Level Phylogeography and Colonization History in River Systems

Delineating basic population units based on past and present genetic connectivity is an important first step in conservation planning. While Arkansas Darters are currently found in the Arkansas River and its tributaries, in the species' current distribution, they are absent from the southern reaches of many of these drainages, suggesting the species may have colonized the entire river system in the past but lacks current connectivity among the drainages in which the genetically defined metapopulations enumerated here are currently found. In a previous species status assessment, stream structure was used to identify a set of metapopulations for the Arkansas Darter (USFWS 2016). Results of genetic analyses presented here provided a means of assessing the validity of these previously described USFWS metapopulation units.

As to be expected for a small stream‐associated fish, stream structure was generally a good predictor of genetic structure. Most drainages previously identified as metapopulations by the USFWS indeed emerged in our analyses as genetically distinct from one another. The USFWS metapopulations with direct stream connections, e.g., most of the Lower Arkansas River and Rattlesnake Creek metapopulations and the North and South Forks of the Ninnescah, usually clustered together. However, this was not always the case. For example, although sites in Slate Creek only have direct hydrological connections with the Lower Arkansas River, they assign strongly with the Ninnescah River genetic deme. Similarly, Salt Fork populations are connected by water to the Medicine Lodge River but are genetically more similar to the Cimarron River. These disparities between stream and genetic structure could represent historic stream capture events, given the complicated hydrological history of Kansas (Fent 1950). Mismatches could also be produced by colonization during flood events, which are particularly common in Great Plains streams and which could allow “lateral” connectivity across drainages (Dodds et al. 2004). Loss of lateral connectivity has exacerbated fragmentation of fish metapopulations in many river systems, and restoration of occasional lateral connectivity, especially when evidence suggests it existed previously, represents a potential means of improving persistence probability (Stoffels et al. 2022). We also observed admixture between the Lower Arkansas and Ninnescah Rivers below the confluence of these two drainages, likely due to downstream dispersal. Regardless of the causes, these divergences from the USFWS metapopulation designations provide important context for understanding patterns of gene flow in this species and guidance for restoring connectivity in the future.

### 4.2 Factors Affecting Genetic Diversity Within and Connectivity Among Populations

Landscape genomics can be a powerful approach for identifying environmental factors that influence genetic diversity and gene flow. Replication of landscape genomic analyses across multiple landscapes is not yet common practice but may be key to robustly inferring associations between environment and genetic differentiation and making useful recommendations for conservation (Keller et al. 2015; Short Bull et al. 2011). Here, we used multiple drainages as replicated units for inferring factors affecting genetic diversity and differentiation. In doing so, we identified substantial differences among drainages in factors related to population density (heterozygosity and the slope of the isolation‐by‐distance relationship), highlighting variability in vulnerability to genetic drift and environmental change among genetically defined metapopulation units for *E. cragini* . After accounting for this variability, however, we found strong evidence that characteristics of streams and their surrounding terrestrial landscapes, as well as modifications to stream flow, also affect gene flow within drainages in a common way, suggesting that targeted conservation actions in specific stream reaches could increase connectivity among small, isolated populations with a high risk of extirpation.

Obstructions to the flow of water (dams and a large reservoir) also emerged as barriers to gene flow in the streamscape genetics model, where they were both associated with increased genetic differentiation. Previous work on darters had found similar effects of dams (Beneteau et al. 2009; Sterling et al. 2013) and reservoirs (Blanton et al. 2024) in some species but also no evidence or equivocal evidence in others (McCall and Fluker 2022; Olsen et al. 2016; Robinson et al. 2013) or species and/or population‐specific effects of dams (Argentina et al. 2018; George et al. 2010; Haponski et al. 2007). Dams may have less of an effect on some darter species inhabiting lower‐order streams if dams are placed mainly on higher‐order streams that would be unsuitable habitat regardless (McCall and Fluker 2022). For *E. cragini* , which are present mainly in tributaries, fragmentation by dams can pose a more significant anthropogenic burden on connectivity. Perhaps fortunately, dams in the study area are mainly located on lower‐order streams near the upstream extent of *E. cragini* habitat occupancy (Figure 1c), meaning that a relatively small number of headwater populations were affected by dams in this study. Dams did not have any detectable effect on heterozygosity, possibly indicating that populations isolated by changes in flow are still large enough or that not enough darter generations have passed to result in strong drift. On the other hand, the Cheney Reservoir, which was constructed from 1962 to 1965, did appear to have a marginal negative effect on heterozygosity, suggesting that genetic drift may already be causing losses of genetic diversity in populations isolated by the inundation of previously suitable streams. In any case, the opportunity for occasional dispersal into populations isolated upstream of current and future dams and reservoirs has likely been entirely lost, and so managers should be aware and proactive of the potential need for assisted migration for maintaining genetic variation and persistence of these populations.

We also found effects of both stream type and upland habitat on genetic differentiation, with more intermittent streams and greater intervening cropland area all associated with greater genetic differentiation and developed land cover associated with decreased genetic differentiation. Water availability had been previously shown to affect connectivity in Colorado populations (Fitzpatrick et al. 2014), and a clear relationship between greater water availability and greater connectivity in perennial streams accords with this finding. Cropland may affect water availability in streams via water diversion to crops. Runoff from crops could also result in increased siltation or pollution with herbicide or insecticide (and thus reduced habitat quality) associated with crops, and darters in agricultural settings have shown adverse responses to agricultural contaminants (Diamond et al. 2016). A negative association between genetic differentiation and developed land cover is somewhat more difficult to explain, and the opposite relationship may be expected as darters have also shown increased stress, higher parasite loads, and altered sexual development in response to contaminants in municipal wastewater (Bahamonde et al. 2015; Diamond et al. 2016; Krause et al. 2010). More developed land cover, however, may simply be associated with lower levels of agricultural development. In addition to pointing to factors to target for riparian habitat restoration, the habitat associations we identified can be factored into decision‐making with respect to which populations to prioritize for translocations. For example, populations separated by high intermittency are presumably good candidate recipient populations for assisted migration. On the other hand, populations surrounded by extensive agriculture may not be worth prioritizing if the water quality is poor.

Notably, we found a positive relationship between upstream distance and genetic diversity. In some other stream species, diversity has been observed to decline with upstream distance due to largely unidirectional downstream dispersal, especially in fish or invertebrates with limited dispersal (Alp et al. 2012; Alther et al. 2021; Delord et al. 2023) and in fish where high grades or waterfalls create strong barriers to upstream dispersal (Deflem et al. 2022; Gouskov and Vorburger 2016; Narum et al. 2008; Torterotot et al. 2014). In the relatively low‐grade streams with fewer barriers to upstream dispersal inhabited by *E. cragini* , however, upstream dispersal may be more common. Research in other darter species has shown weak or stream‐specific relationships between upstream distance and genetic diversity (Euclide and Marsden 2018; Luiken et al. 2021) and no strong bias toward downstream versus upstream dispersal (Beneteau et al. 2009). Arkansas darters, in particular, tend to have higher abundances in more shallow and narrow stream reaches (Wellemeyer et al. 2019), and as such, a positive correlation between upstream distance could reflect higher population densities in headwater reaches of inhabited streams. These upstream populations may therefore be important reservoirs of diversity for the species.

The slope of the isolation‐by‐distance relationship (Figure 2) varied substantially among drainages and was highest in the Salt Fork as well as two genetically defined metapopulations in Colorado (Big Sandy/Rush Creeks and the Middle Arkansas). Variation in the relationship between distance and genetic differentiation aligns with theory, which predicts the slope of isolation‐by‐distance should be inversely proportional to effective population size (Rousset 1997). The third Colorado metapopulation (Upper Arkansas) with a relatively small effective population size, had a low slope but a large intercept for the IBD relationship, suggesting all sites were highly diverged from one another regardless of distance. IBD was not evaluated for the drainage with the smallest effective size (Walnut Creek), which only contained three sampled sites. In all of these genetically defined metapopulations, very strong IBD suggests little genetic or demographic connectivity, indicating these metapopulations should be prioritized for consideration of assisted migration to restore connectivity and maintain variation within these isolated populations.

Metrics of genetic diversity for the Eastern genetically defined metapopulations (Illinois River and Spring River populations) based on Rapture data and whole‐genome sequencing conflicted somewhat; heterozygosity for Rapture loci were extremely low, but estimates of whole‐genome genetic diversity for one individual from the Spring River were the highest among all genetically defined metapopulations. Previous work had identified these populations as outgroups to the other Kansas and Colorado populations that had diverged potentially several million years ago (Reid et al. 2021), suggesting potential species‐level divergence between Arkansas drainages and Ozark drainages. It is possible that low heterozygosity at Rapture loci for these populations may be an artifact of the Rapture probe design process, which used mostly individuals from the Western populations and may thus have selected for loci that are heterozygous in these drainages but not in outgroups.

### 4.3 Relatively Low Levels of Inbreeding and Deleterious Variation

Arkansas darter populations vary widely in levels of heterozygosity and in estimated effective population size. Although we were only able to estimate effective population size for a subset of populations sampled here, several of our effective size estimates were below predicted lower boundaries for short‐term population viability in the face of inbreeding (*N*_e_ < 100) and most point estimates were below the predicted lower boundaries for long‐term maintenance of evolutionary potential (*N*_e_ < 1000; Frankham et al. 2014). Estimates of effective size for Colorado and Arkansas populations from previous studies are even lower than those estimated for Kansas populations here (Fitzpatrick et al. 2014; Baker et al. 2018). Even in populations with very low estimated effective population sizes and low heterozygosity, however, we did not see strong evidence of recent, severe inbreeding in the form of long runs of homozygosity. This suggests that population declines may have been recent enough that inbreeding has yet to become a problem. There may also be mechanisms for avoiding mating with close relatives within darter populations that reduce the frequency of inbreeding, as has been documented in other fish (Ala‐Honkola et al. 2010; Frommen and Bakker 2006), and polygynous darter mating behaviors may increase the number of effective breeders as well (DeWoody et al. 2000). As the LD‐based estimates of *N*_e_ provided here and in other reports apply to subdivided metapopulations, we note that they may underestimate effective size as it relates to inbreeding and adaptive variance (Ryman et al. 2019).

We also did not see particularly high levels of deleterious variation in small populations with low overall heterozygosity. This is somewhat surprising considering that selection should not be able to efficiently remove deleterious variants in small populations. In other fish, smaller populations have been associated with increased deleterious variation (Perrier et al. 2017). However, other studies have also found little deleterious variation in some species with small contemporary effective population sizes (Robinson et al. 2022). Strong recent bottlenecks may result in the purging of highly deleterious mutations but the accumulation of mildly deleterious mutations (Grossen et al. 2020), and persistence at small population sizes over long periods of time may result in the purging of deleterious variation as well (Robinson et al. 2018). Purging over longer timescales in small populations of *E. cragini* during the colonization of western drainages may have prevented the accumulation of deleterious variation, as purging through founder effects has also been noted in populations of several endangered species (Grossen et al. 2020; Mathur, Mason, et al. 2023; Mathur, Tomeček, et al. 2023). While these past studies and the evidence presented here suggest that some populations may be temporarily buffered against the problems that plague small populations, we note that few studies truly measure fitness in small populations over long time scales and that these small populations are likely still more vulnerable to both intrinsic and extrinsic factors that could push them toward extinction.

### 4.4 Adaptive and Structural Variation

Maintaining adaptive variance should also be a priority for management. After accounting for population structure, we found little evidence for associations between the Rapture loci and environmental variation across the range of the Arkansas darter. This result confirms that these randomly selected loci were mainly selectively neutral but should not be taken as evidence that darters are not adapted to local conditions, and we anticipate that additional work with more whole‐genome data could potentially uncover evidence of local adaptation.

Structural variation in genomes and the architecture of local adaptation can also play an important role in both evolutionary outcomes and the outcomes of translocations. Chromosomal inversions are particularly important for preserving local adaptation when gene flow across environmental gradients is high (Wellenreuther et al. 2019). In freshwater fish species with more restricted gene flow, however, genetic variants under selection tend to be more evenly distributed throughout the genome (Shi et al. 2023). Given the strong isolation observed among genetically defined metapopulations, we expect that locally adapted loci would likely be more evenly distributed throughout the genome rather than clustered within inverted regions. In this case, genome‐wide diversity may be the most reasonable proxy for preserving adaptive variation (Kardos et al. 2021). Chromosomal differences, particularly duplications, translocations, and inversions, are also associated with greater outbreeding depression (Frankham et al. 2011). We found evidence for numerous structural variants (primarily small insertions and deletions, but also potential inversions, translocations, and duplications) among Arkansas darter populations. While indels and duplications are unlikely to cause outbreeding depression (Frankham et al. 2011), the frequency of these variants followed the same pattern across genetically defined metapopulations as breakend variants associated with inversions and translocations, suggesting that indels may represent a proxy for these more potentially deleterious variants. Acquiring more long‐read genetic data would be useful for better characterizing these variants, given the limitations of short‐read data for detecting larger variants (Mahmoud et al. 2019), and determining whether they are fixed in populations.

### 4.5 Are Arkansas Darters in Need of Assisted Migration?

Not all small populations with low genetic diversity are destined to succumb to the extinction vortex. However, there are several compelling reasons to suggest that, despite lacking strong evidence for recent inbreeding or accumulation of deleterious variation, some populations throughout the range of the Arkansas darter would benefit from restoring connectivity through assisted migration. This Great Plains‐adapted species is thought to persist via classic metapopulation dynamics that rely on occasional dispersal among populations to maintain genetic integrity and to recolonize areas that have been extirpated by dewatering or drought. Barriers to dispersal and gene flow such as stream intermittency (which is expected to worsen) and dams, reservoirs, and other impoundments preclude successful dispersal of Arkansas darters within and among metapopulations. Our landscape genetic analyses indeed revealed that these factors, and others, contribute to isolation among darter populations. These findings are paired with exceedingly low genetic variation, small effective population sizes, and steep demographic declines in some portions of the range of *E. cragini* . Given the low natural dispersal ability of this species and multiple, compounding factors leading to increased isolation, including climate warming, surface and groundwater removal, physical barriers, and habitat degradation, consideration of assisted migration to augment and restore connectivity among certain populations is strongly recommended. Populations with low variation and small effective sizes are limited in their capacity to respond via adaptation to changing environmental conditions, further increasing their risk of extirpation through stochastic or deterministic processes (Kardos et al. 2021). A key point is that carrying out rangewide genetic assessments and considering assisted migration strategies while options still exist both (1) provides managers with the ability to prioritize among populations and (2) should improve the likelihood of successful outcomes. Below we discuss a general framework for leveraging population and landscape genomic datasets to balance inbreeding and outbreeding risks when evaluating assisted migration strategies while also providing specific recommendations for *E. cragini*.

**FIGURE 6:**
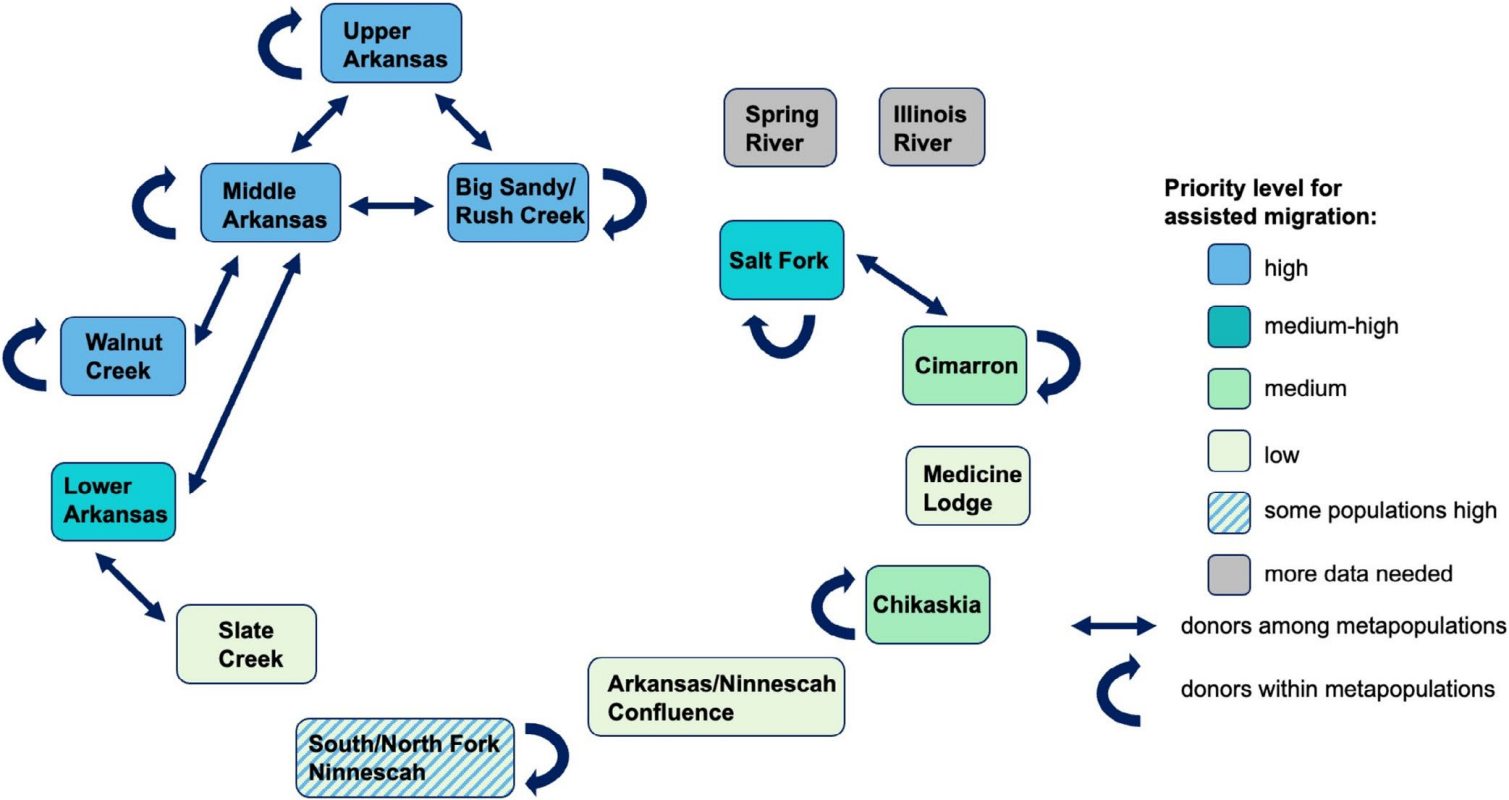
Potential donor and recipient populations for assisted gene flow. Colors indicate the priority level for assisted migration; straight lines indicate that assisted migration among genetically defined metapopulations is possible, while curved lines indicate that assisted migration among sites within a metapopulation could be ideal.

The rangewide landscape genomic data presented here can be used to extract multiple lines of evidence for informing and prioritizing assisted migration strategies that would most benefit the species. First, populations facing the most severe “small population problems” can be identified. These are presumably the populations with the highest extirpation risk and thus stand to benefit the most from new genetic variation provided by gene flow. In theory, these “high‐risk” populations would show relatively low levels of neutral genetic diversity and heterozygosity, high levels of deleterious variation, inbreeding, and isolation, and small effective population sizes (Table 1). Despite a lack of evidence for recent inbreeding, our dataset suggests that at the rangewide level, populations in the western part of the range (i.e., all genetically defined metapopulations in Colorado and the Walnut Creek metapopulation in Kansas) are the most vulnerable to extirpation due to genetic and demographic factors. Within Kansas, darter populations in the Lower Arkansas and Salt Fork Arkansas River metapopulations would be a second priority to target with assisted migration due to relatively low heterozygosity and effective population size and high pairwise F_ST_s, suggesting small overall population size and isolation (Figure 6). Finally, populations from the North Fork of the Ninnescah River inhabiting tributaries upstream of the Cheney Reservoir show evidence of recent isolation due to the creation of this impoundment, although genetic diversity is still relatively high within these populations. Given the man‐made barrier to connectivity among these sites, these populations should be considered as eventual targets for assisted migration to ensure long‐term persistence and adaptive potential.

Once target recipient populations have been identified, the same genomic datasets can be used to identify potential donor populations (or even individuals) that would minimize the risk of outbreeding depression. Ideal donor populations should have medium to low levels of differentiation from the recipient population and low deleterious variation, inbreeding, and structural variants relative to the recipient (Table 1). Whether donor individuals should be sourced from populations with relatively high or low levels of neutral genetic diversity is an ongoing debate. There is an argument, based on sound population genetic theory, that historically small donor populations have more effectively purged deleterious variation and would thus be safer than sourcing from a historically large donor population (Kyriazis et al. 2021). However, this argument does not take into account several other important theoretical considerations, such as the role of overdominance or heterozygote advantage, and the importance of increased genetic variation for an effective response to selection. Given that many populations face increasingly stressful and fast‐changing environments, this added benefit of increased genome‐wide genetic variation is likely increasingly important (Kardos et al. 2021). Additionally, assisted migration for genetic rescue is typically only discussed when populations have become recently small and isolated due to human impacts (i.e., within the last 200 years). The reality not taken into account by recent simulation studies is that many small populations have likely not had sufficient time to purge genetic load. If options exist, we recommend sourcing from medium or large donor populations.

For Arkansas Darters, given the strong divergence among genetically defined metapopulations, the safest donor populations would be those within the same metapopulation as the recipients. Alternatively, relatively safe donor populations from other metapopulations can be identified based on our data. For example, Walnut Creek and Colorado populations show low levels of structural variation relative to one another and to the Lower Arkansas and Rattlesnake metapopulations. Coupled with relatively recent estimated divergence times among these metapopulations (Reid et al. 2021), these populations likely share a recent common ancestor, and as such, the risk of outbreeding depression between these populations is likely low. The Salt Fork Arkansas River metapopulation also shows a strong affinity to the Cimarron River metapopulation, although the two do not share a direct water connection (possibly due to river capture), and assisted gene flow from Cimarron populations to the Salt Fork could also be an option that minimizes potential outbreeding depression. Individuals sourced from populations downstream of the Cheney Reservoir could be used to infuse genetic variation and ameliorate genetic drift in the recently isolated upstream populations.

Postrescue monitoring is an important component of genetic rescue, as continued observation can clarify how genomic diversity and population trends change in response to assisted gene flow (Fitzpatrick and Funk 2021). Establishing baselines for genomic diversity and census sizes for recipient populations prior to receiving translocated individuals is a crucial first step in this monitoring, and using whole‐genome sequencing to fully characterize nucleotide diversity, genome‐wide differentiation, genetic load, and structural variants in both donor and recipient populations before translocations would be key to evaluating the success of translocations in increasing genetic diversity. After translocations occur, populations should be monitored for both overall population‐level diversity and for the presence of F1 and backcross hybrids to confirm successful outbreeding of resident individuals with migrants. As the initial benefits of rescue accrue over at least the first three generations after rescue (Frankham 2016), postrescue monitoring of demography, genetic load, and fitness parameters could be conducted at least every three generations (or more frequently depending on resources available). If translocated individuals are highly successful relative to residents, potentially concerning outcomes include swamping of local ancestry and/or high reproductive skew leading to eventual genetic erosion within the targeted population. These outcomes can be detected whenever population genetic data from at least two time points permit calculating relatedness between ancestors and descendants (Linderoth et al., 2025). After initial monitoring, additional translocations can be performed to further augment genetic diversity if initial attempts fail to produce outbred individuals, or if high drift and inbreeding start to accumulate once again, or if high reproductive skew is detected.

## 5 Conclusions

Habitat loss and fragmentation are two of the most common drivers of biodiversity loss, and an underused strategy for mitigating the effects of these is human‐assisted migration. Inevitably, there are many uncertainties and risks involved with carrying out assisted migration, although ‘doing nothing’ may often be just as risky. Our study highlights how modern genomic tools can be leveraged to minimize risk and serves as a model for applying landscape genomics to the conservation and management of recently fragmented populations. The ability to identify recipient populations of greatest concern and to choose from multiple potential donor populations, based on detailed genetic information, should improve the likelihood of a beneficial outcome. Given the extent of the biodiversity crisis, these well‐informed human interventions to species conservation are increasingly vital.

## Conflicts of Interest

The authors declare no conflicts of interest.

## Supporting information


**Figure S1.** (a) Environmental gradients across the study area. Each colored dot shows the interpolated value for a given environmental variable at each study site. Warmer colors represent relatively high values, while cooler colors represent relatively low values. (b) LFMM *p* values for each SNP × environmental variable combination. Dotted line indicates threshold for statistical significance after Bonferroni‐Holm correction.

**Figure S2.** Barplot showing proportion missing Rapture genotypes averaged across individuals for each genetically defined metapopulation.

**Figure S3.** PCA results showing the first 20 principal component axes for the Rapture dataset. Each genetically defined population is shown in a distinct color, with individuals shown as dots and confidence ellipses. Percent variation explained is shown on each principal component axis.

**Figure S4.** Cross‐entropy statistic calculated by snmf for the number of ancestral populations (*k*) from 2 to 20.

**Figure S5.** (a) Heat map of pairwise *F*_ST_ values between each pair of USFWS‐defined metapopulations. (b) Heat map of pairwise *F*_ST_ values between each pair of genetically defined metapopulations.

**Figure S6.** Isolation‐by‐distance in North Fork Ninnescah River. Pairwise *F*_ST_ values for which at least one pair occupies an isolated tributaries to the Cheney Reservoir are shown in gray circles, while sites for which both neither pair are in an isolated tributary are shown in blue. Fitted relationships between *F*_ST_ and distance are shown for either group by gray and blue lines, respectively.

**Figure S7.** Distribution of random *R* ^2^ values (gray histogram) compared to *R* ^2^ from best model (red dotted line) for the landscape genetics analysis.

**Figure S8.** Relationship between whole‐genome heterozygosity and average Rapture heterozygosity for genetically defined metapopulations to which each WGS individual belongs.

**Figure S9.** Density plot showing distribution of insertion and deletion sizes across different genetically defined metapopulations.

**Figure S10.** Number of breakend variants (upper plots) and duplications (lower plots) identified by Manta, plotted against either divergence time (right) or *F*_ST_ (left) compared to Colorado populations.

**Table S1.** Site locations, sample sizes, and heterozygosities for Rapture dataset.

**Table S2.** Individual information for WGS dataset.

**Table S3.** (a) Average *F*_ST_ values for each genetically defined metapopulation. (b) Pairwise *F*_ST_ values among genetically defined metapopulations with bootstrap confidence intervals.

**Table S4.** Model selection table for heterozygosity analysis.

**Table S5.** Model selection table for *F*_ST_ analysis.

## Data Availability

The *E. cragini* Whole Genome Shotgun project is available at DDBJ/ENA/GenBank under accession JAAVJE000000000. The version described in this paper is version JAAVJE010000000. Short‐read data have been uploaded to the NCBI as BioProject PRJNA611833. Analysis scripts and metadata are available at https://github.com/nerdbrained/darter_streamscape_genomics.
