# Supplementary figures and images for: Balancing Inbreeding and Outbreeding Risks to Inform Translocations Throughout the Range of an Imperiled Darter
Source: Evol Appl. 2025 Mar 23;18(3):e70088. doi: 10.1111/eva.70088 (PMC11930765; doi:10.1111/eva.70088)

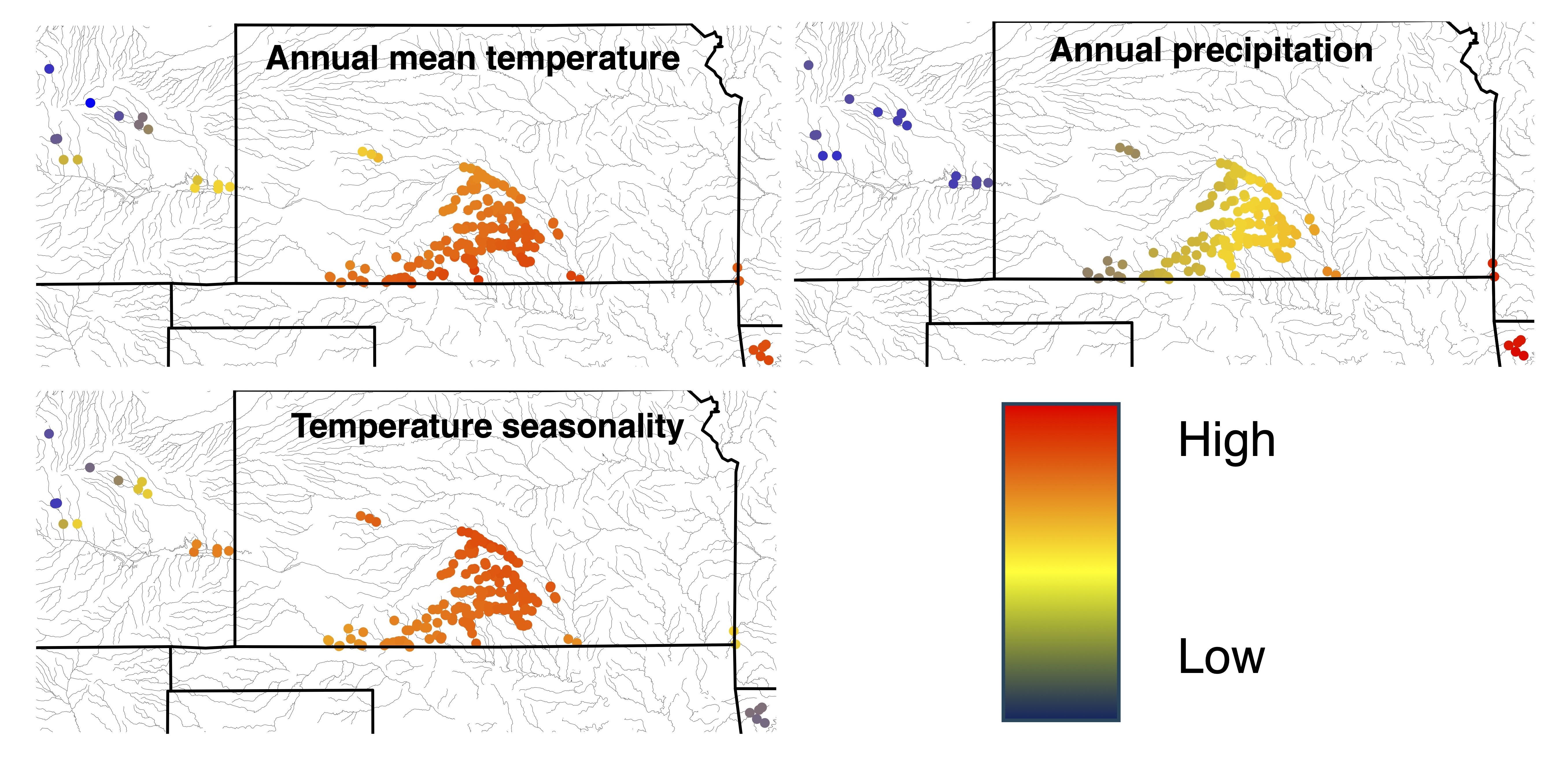

Supplement: Supplementary file 1 — Figure S1. (a) Environmental gradients across the study area. Each colored dot shows the interpolated value for a given environmental variable at each study site. Warmer colors represent relatively high values, while cooler colors represent relatively low values. (b) LFMM p values for each SNP × environmental variable combination. Dotted line indicates threshold for statistical significance after Bonferroni‐Holm correction. [file EVA-18-e70088-s014.zip › EVA70088-sup-0002-supinfo_SuppFigure1a_envivars.jpg]

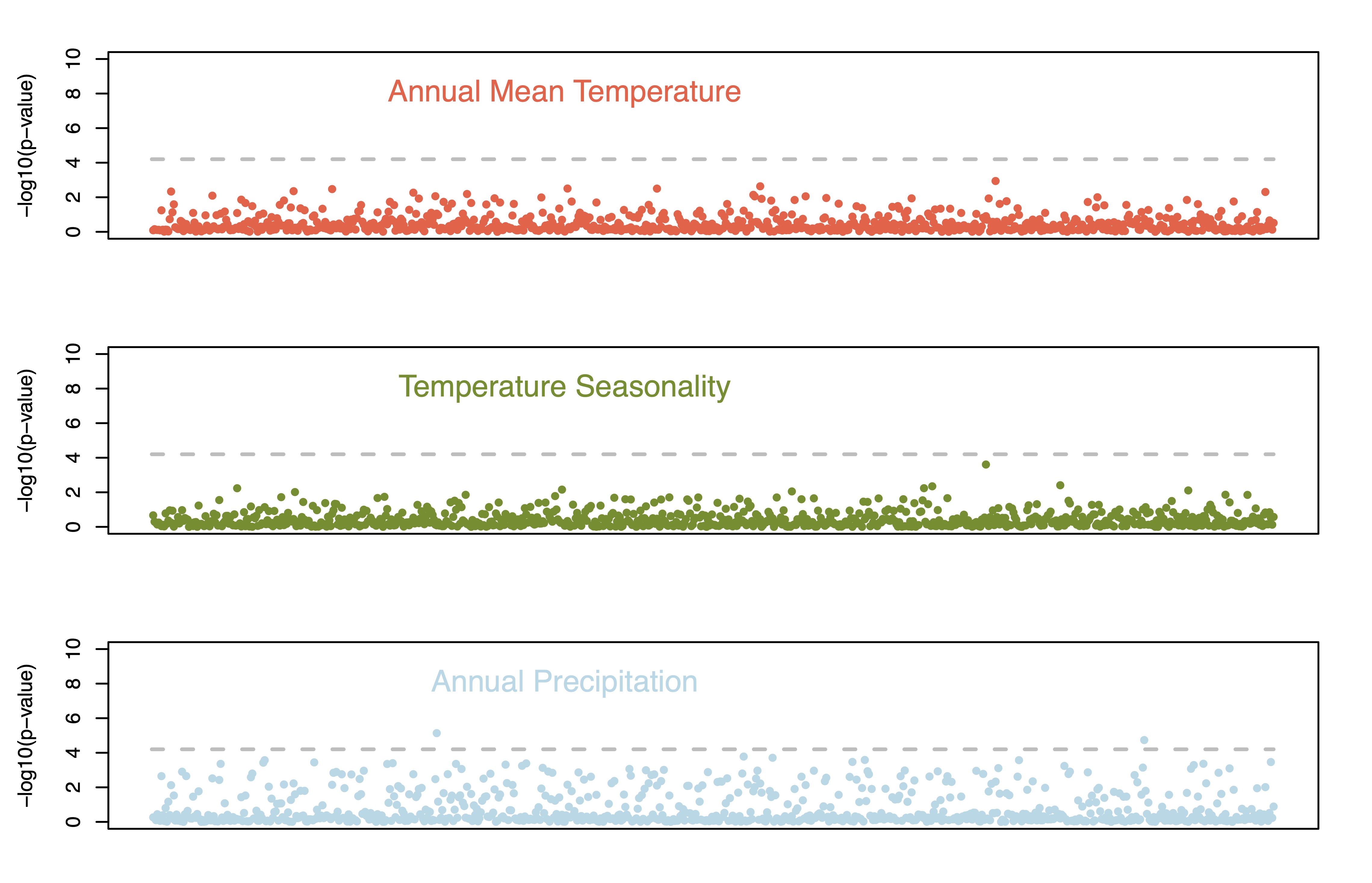

Supplement: Supplementary file 1 — Figure S1. (a) Environmental gradients across the study area. Each colored dot shows the interpolated value for a given environmental variable at each study site. Warmer colors represent relatively high values, while cooler colors represent relatively low values. (b) LFMM p values for each SNP × environmental variable combination. Dotted line indicates threshold for statistical significance after Bonferroni‐Holm correction. [file EVA-18-e70088-s014.zip › EVA70088-sup-0003-supinfo_SuppFigure1b_lfmm.jpg]

Proportion missing data for Rapture loci per metapopulation

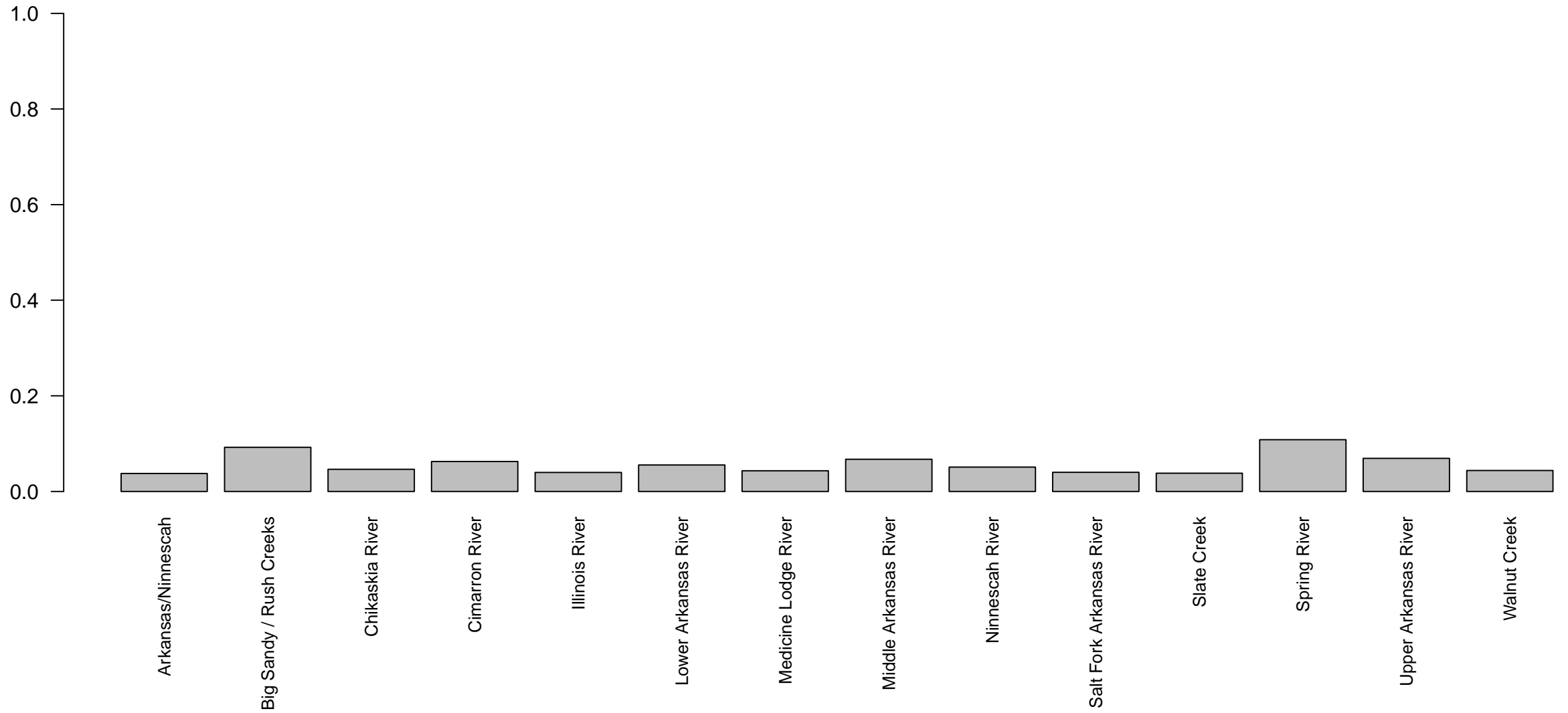

Supplement: Supplementary file 2 — Figure S2. Barplot showing proportion missing Rapture genotypes averaged across individuals for each genetically defined metapopulation. [file EVA-18-e70088-s009.pdf]

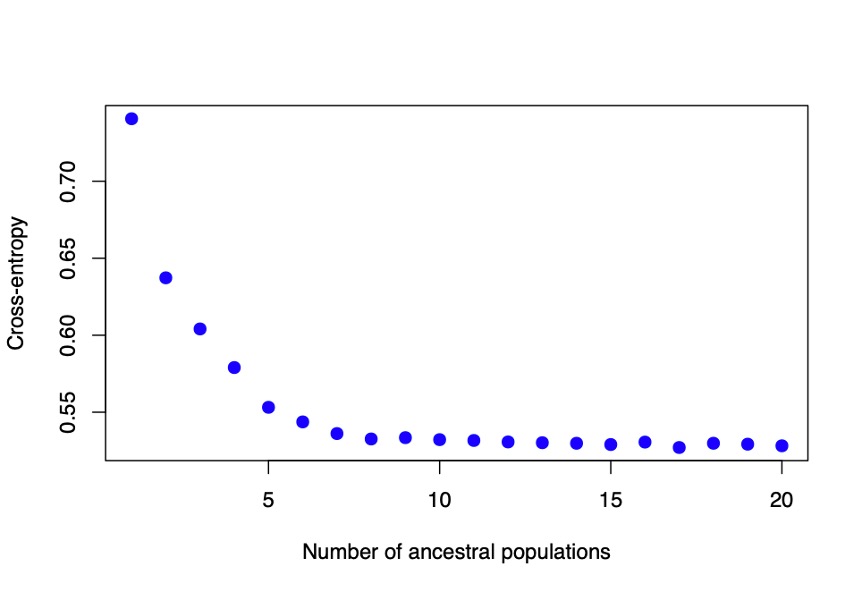

Supplement: Supplementary file 4 — Figure S4. Cross‐entropy statistic calculated by snmf for the number of ancestral populations (k) from 2 to 20. [file EVA-18-e70088-s011.jpg]

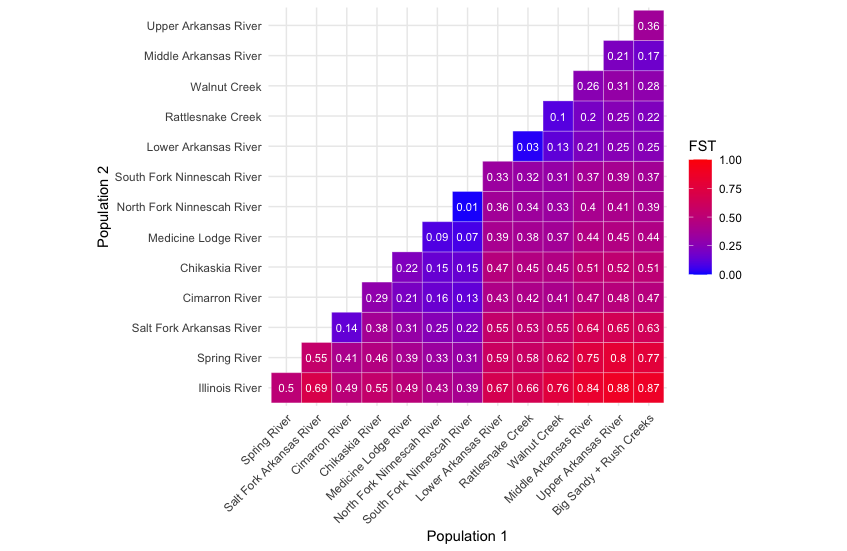

Supplement: Supplementary file 5 — Figure S5. (a) Heat map of pairwise F ST values between each pair of USFWS‐defined metapopulations. (b) Heat map of pairwise F ST values between each pair of genetically defined metapopulations. [file EVA-18-e70088-s001.zip › eva70088-sup-0005-FigureS5a.png]

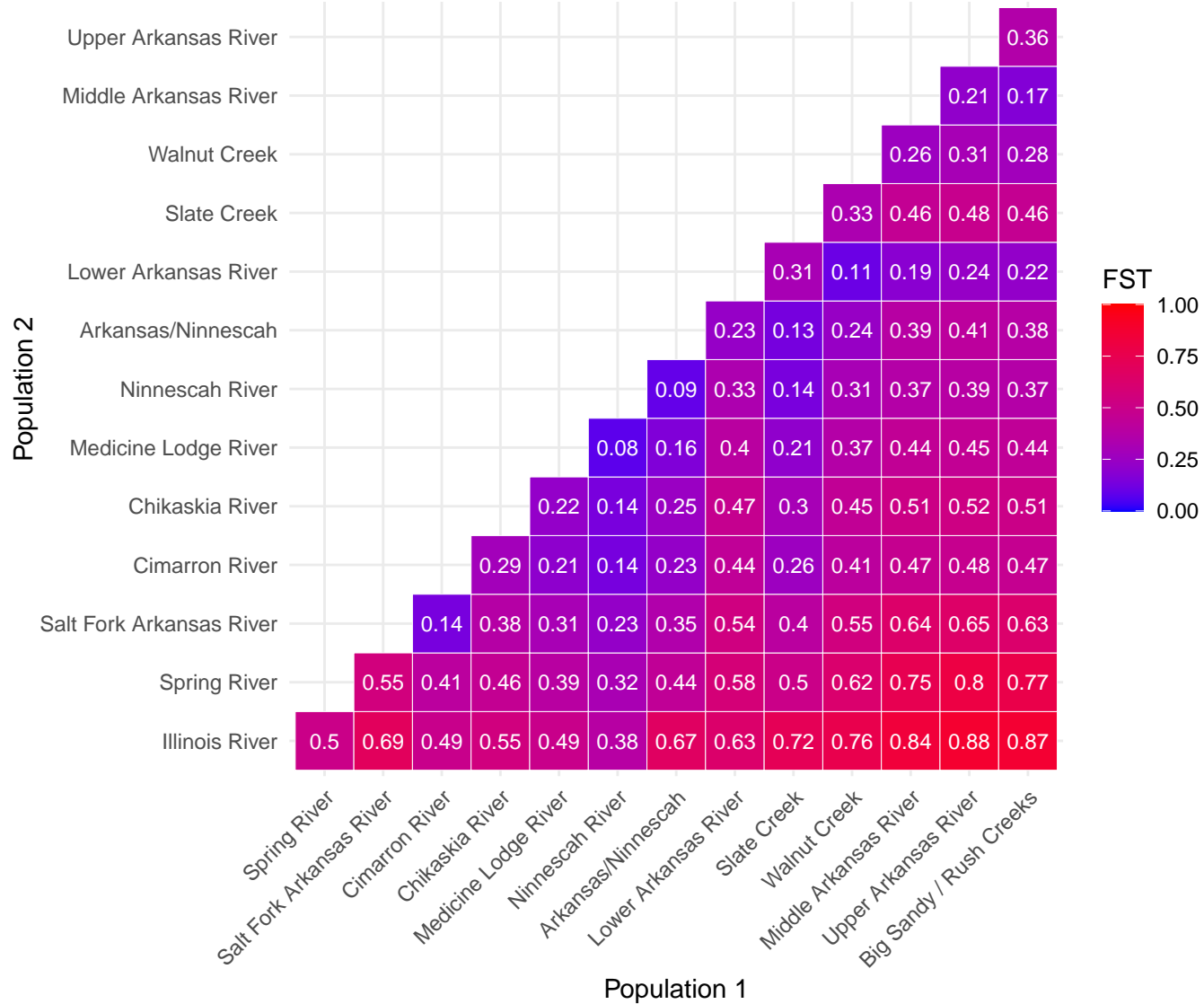

Supplement: Supplementary file 5 — Figure S5. (a) Heat map of pairwise F ST values between each pair of USFWS‐defined metapopulations. (b) Heat map of pairwise F ST values between each pair of genetically defined metapopulations. [file EVA-18-e70088-s001.zip › eva70088-sup-0006-FigureS5b.pdf]

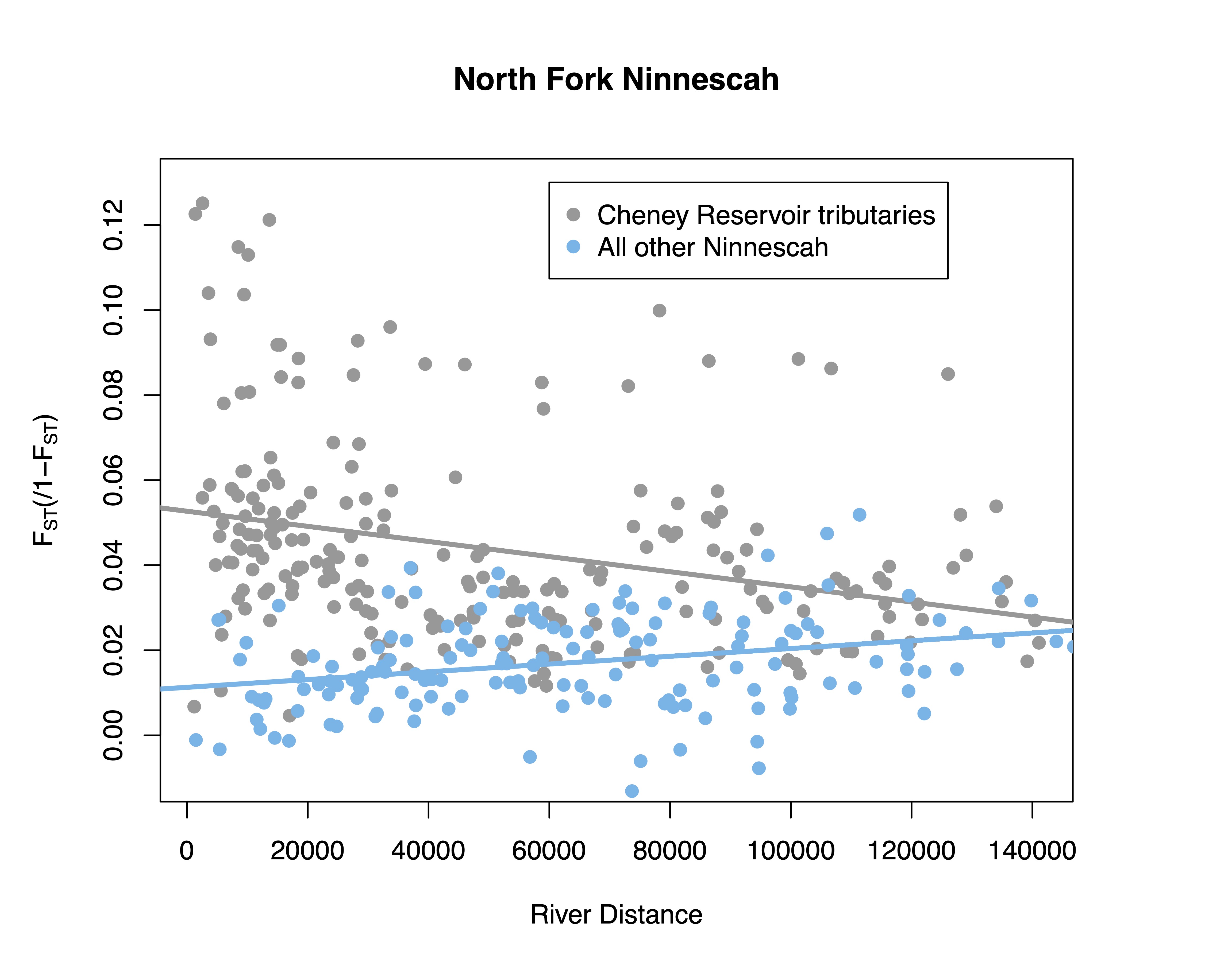

Supplement: Supplementary file 6 — Figure S6. Isolation‐by‐distance in North Fork Ninnescah River. Pairwise F ST values for which at least one pair occupies an isolated tributaries to the Cheney Reservoir are shown in gray circles, while sites for which both neither pair are in an isolated tributary are shown in blue. Fitted relationships between F ST and distance are shown for either group by gray and blue lines, respectively. [file EVA-18-e70088-s004.jpg]

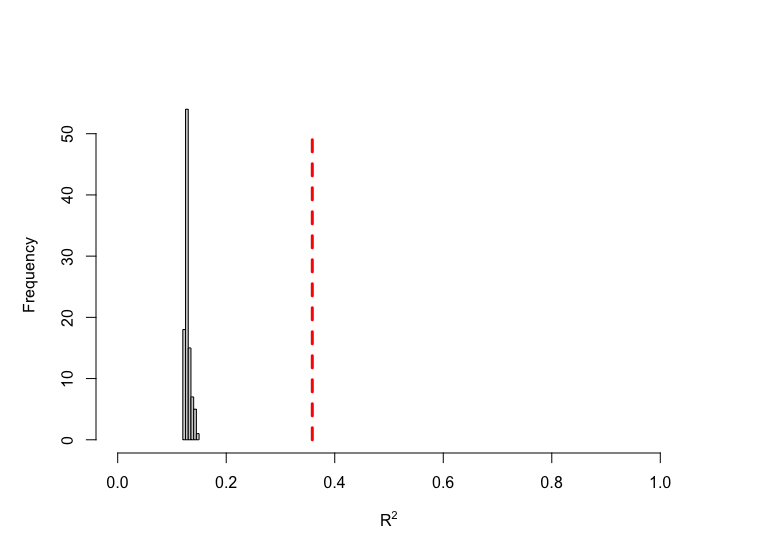

Supplement: Supplementary file 7 — Figure S7. Distribution of random R 2 values (gray histogram) compared to R 2 from best model (red dotted line) for the landscape genetics analysis. [file EVA-18-e70088-s005.png]

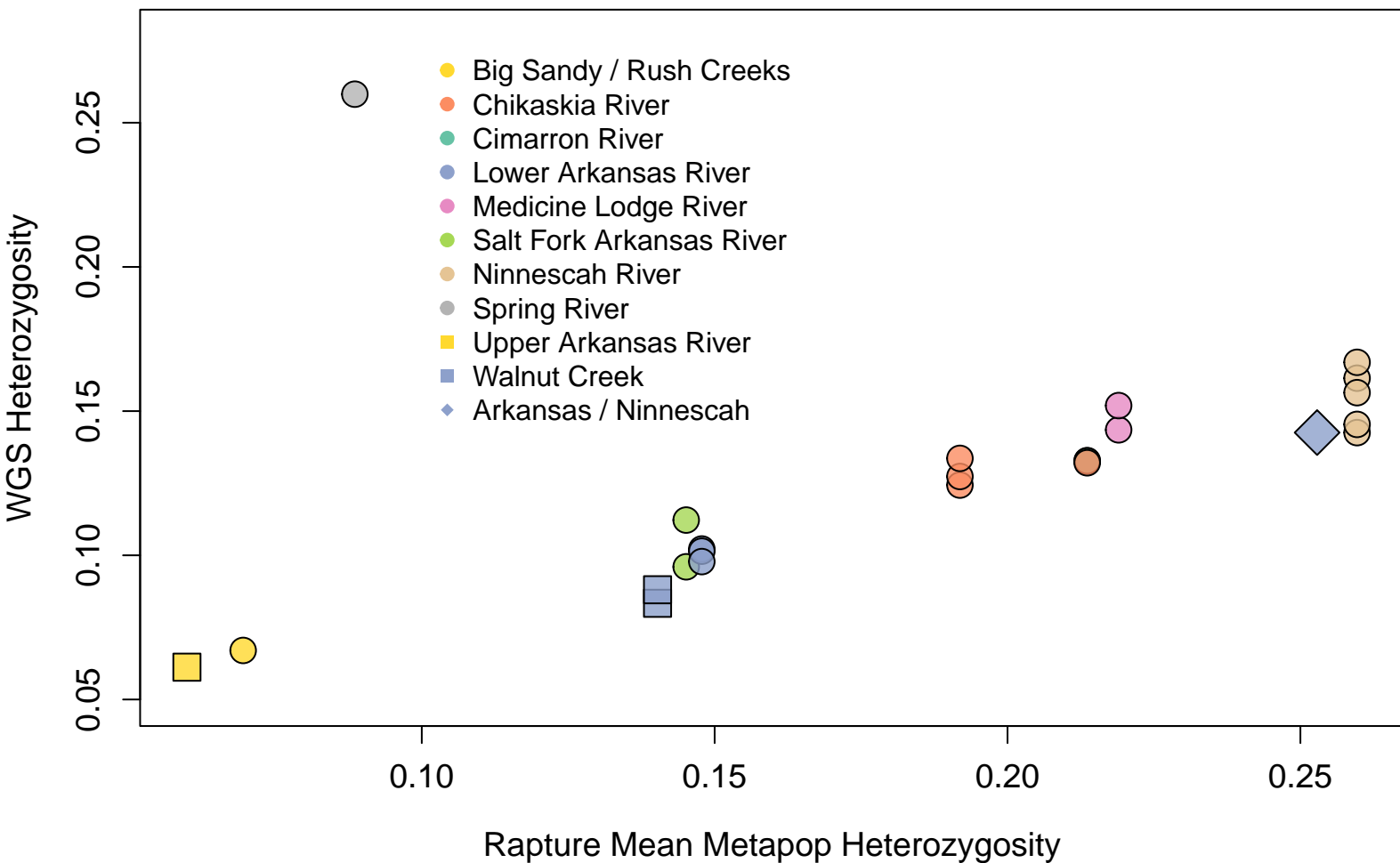

Supplement: Supplementary file 8 — Figure S8. Relationship between whole‐genome heterozygosity and average Rapture heterozygosity for genetically defined metapopulations to which each WGS individual belongs. [file EVA-18-e70088-s015.pdf]

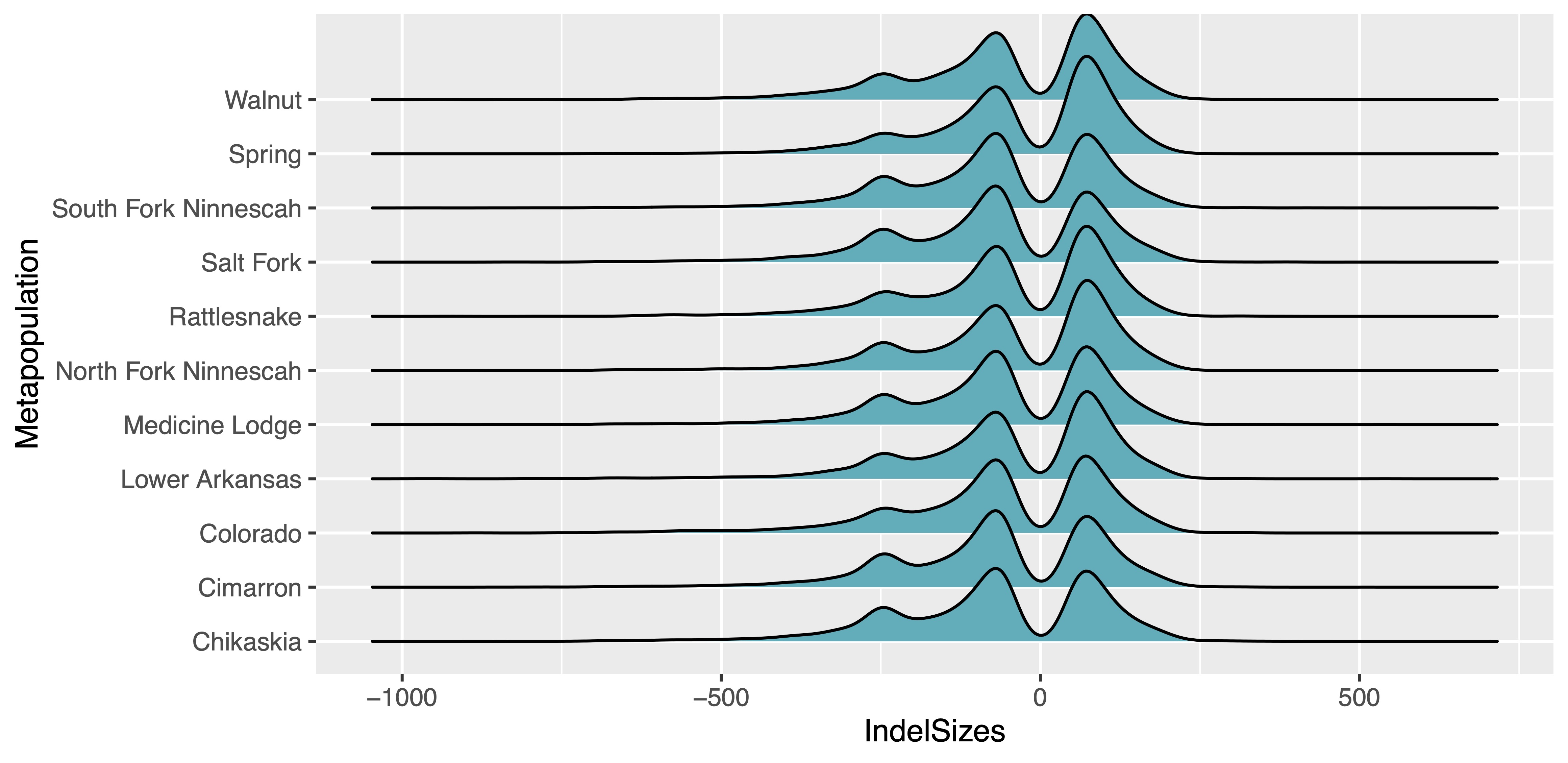

Supplement: Supplementary file 9 — Figure S9. Density plot showing distribution of insertion and deletion sizes across different genetically defined metapopulations. [file EVA-18-e70088-s006.jpeg]

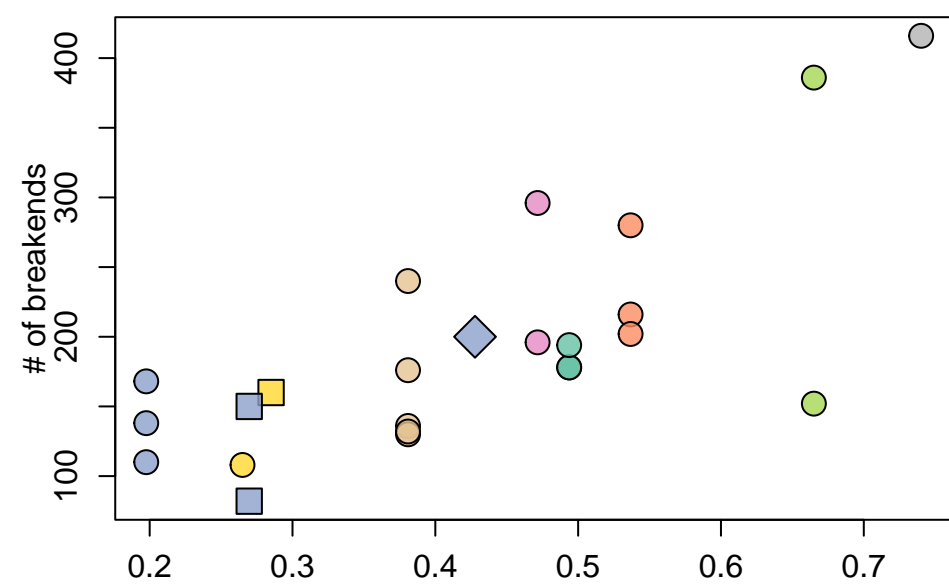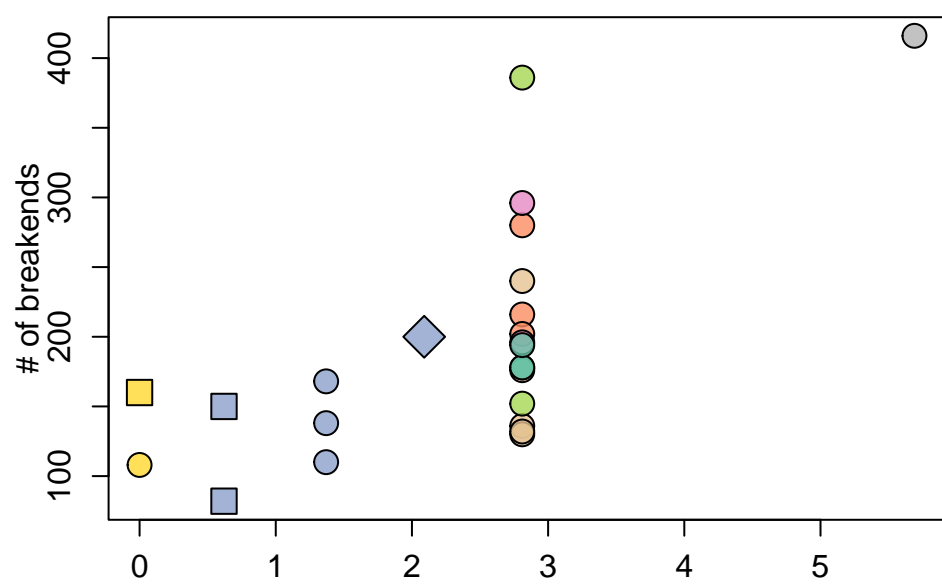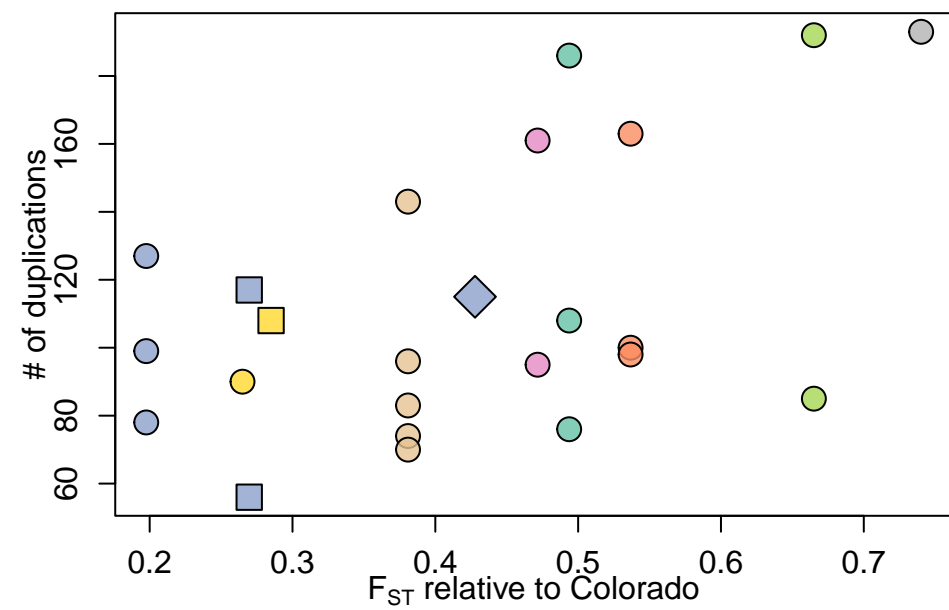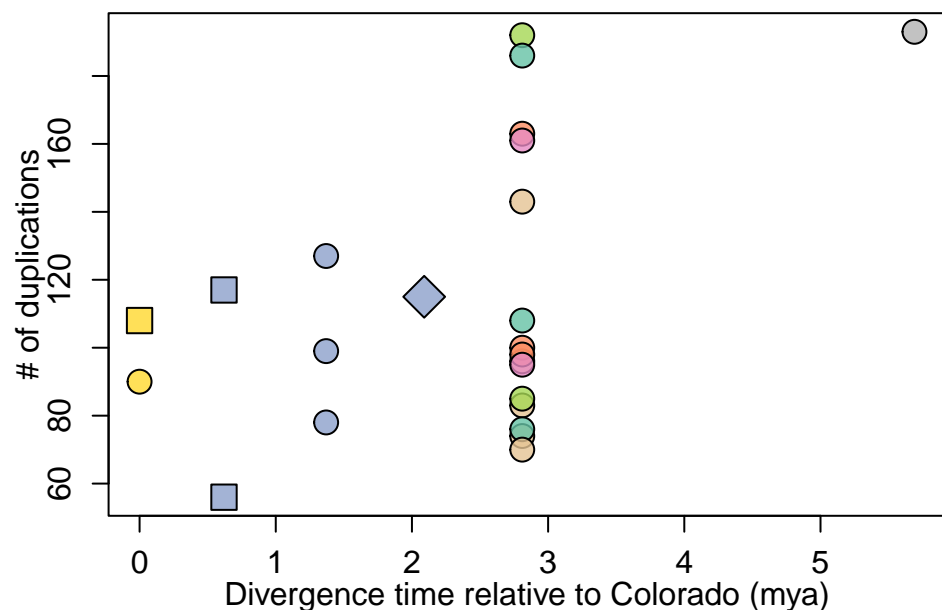

Supplement: Supplementary file 10 — Figure S10. Number of breakend variants (upper plots) and duplications (lower plots) identified by Manta, plotted against either divergence time (right) or F ST (left) compared to Colorado populations. [file EVA-18-e70088-s012.pdf]
