# Supplementary material for: Balancing Inbreeding and Outbreeding Risks to Inform Translocations Throughout the Range of an Imperiled Darter
Source: Evol Appl. 2025 Mar 23;18(3):e70088. doi: 10.1111/eva.70088 (PMC11930765; doi:10.1111/eva.70088)

Supplemental Figure 3a. Principal components analysis (PC1 + PC2)

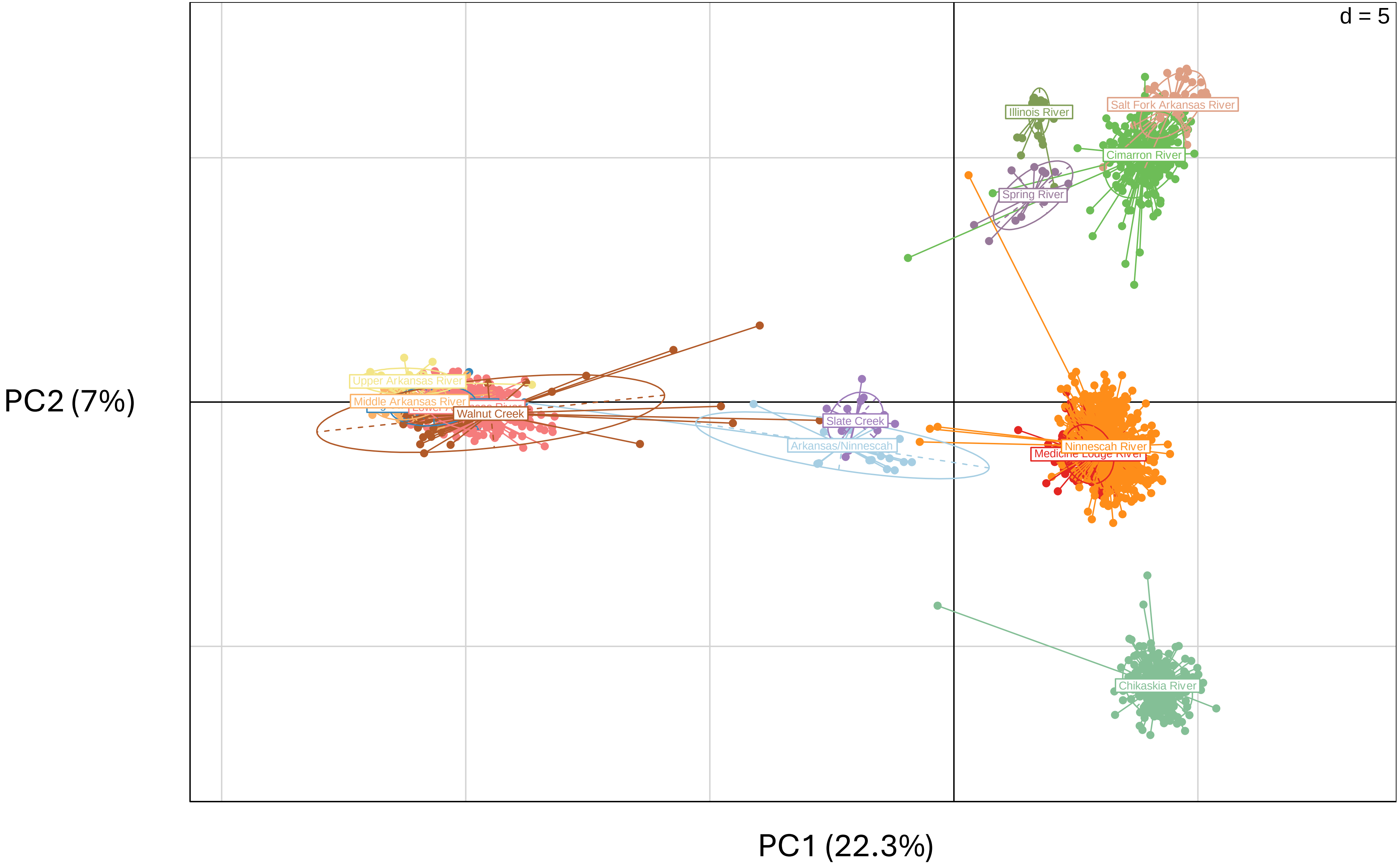

Supplemental Figure 3b. Principal components analysis (PC3 + PC4)

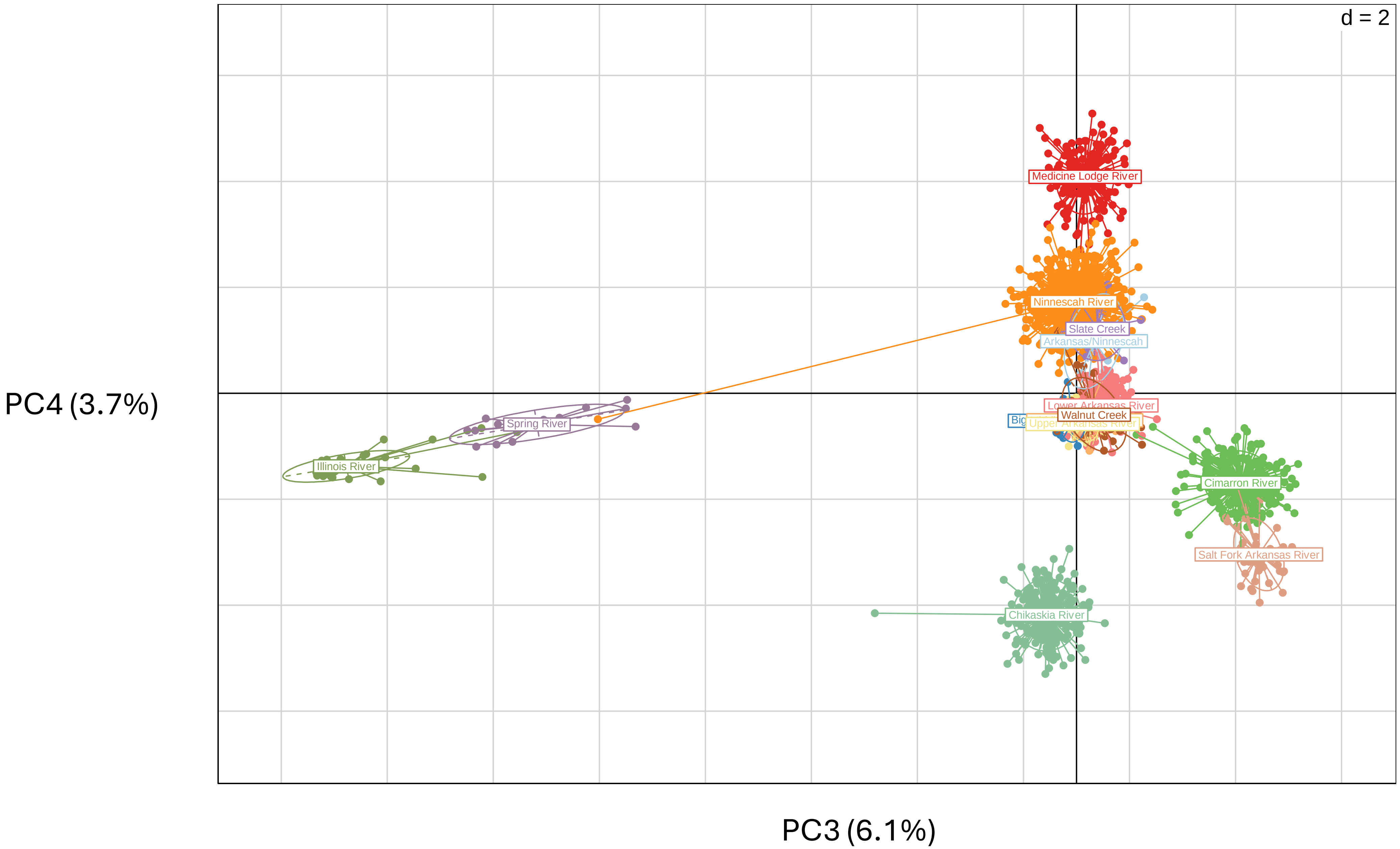

Supplemental Figure 3c. Principal components analysis (PC5 + PC6)

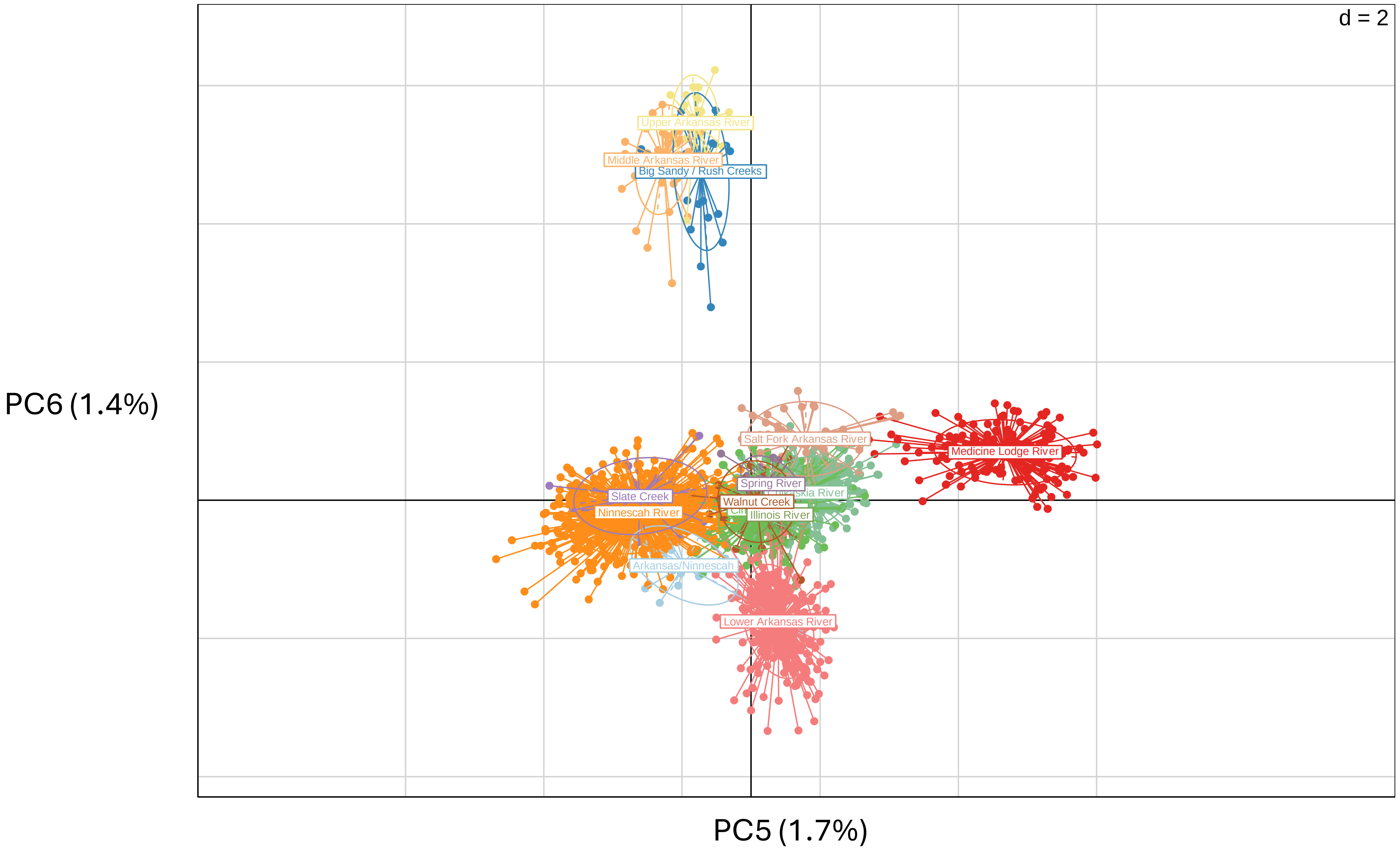

Supplemental Figure 3d. Principal components analysis (PC7 + PC8)

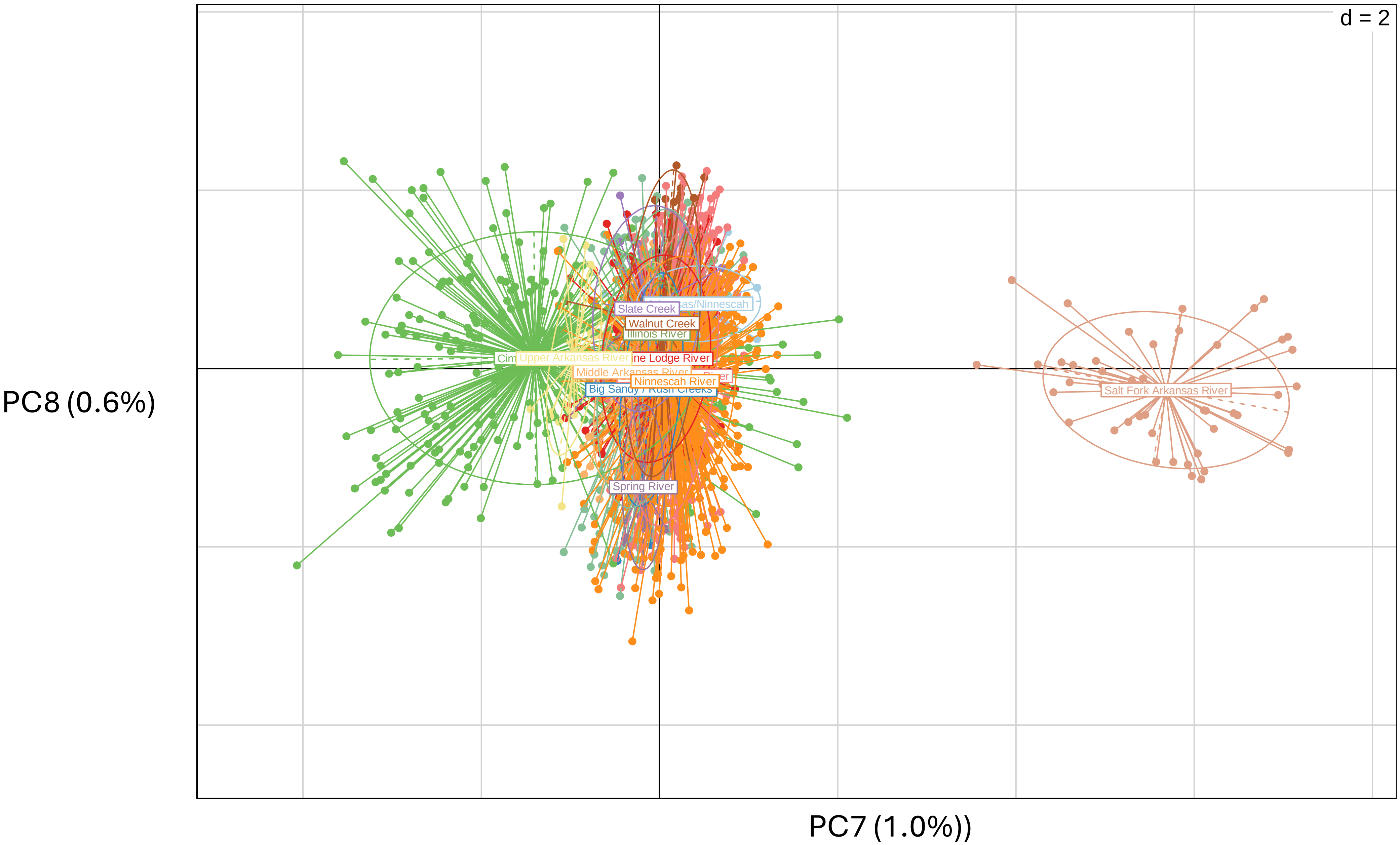

Supplemental Figure 3e. Principal components analysis (PC9 + PC10)

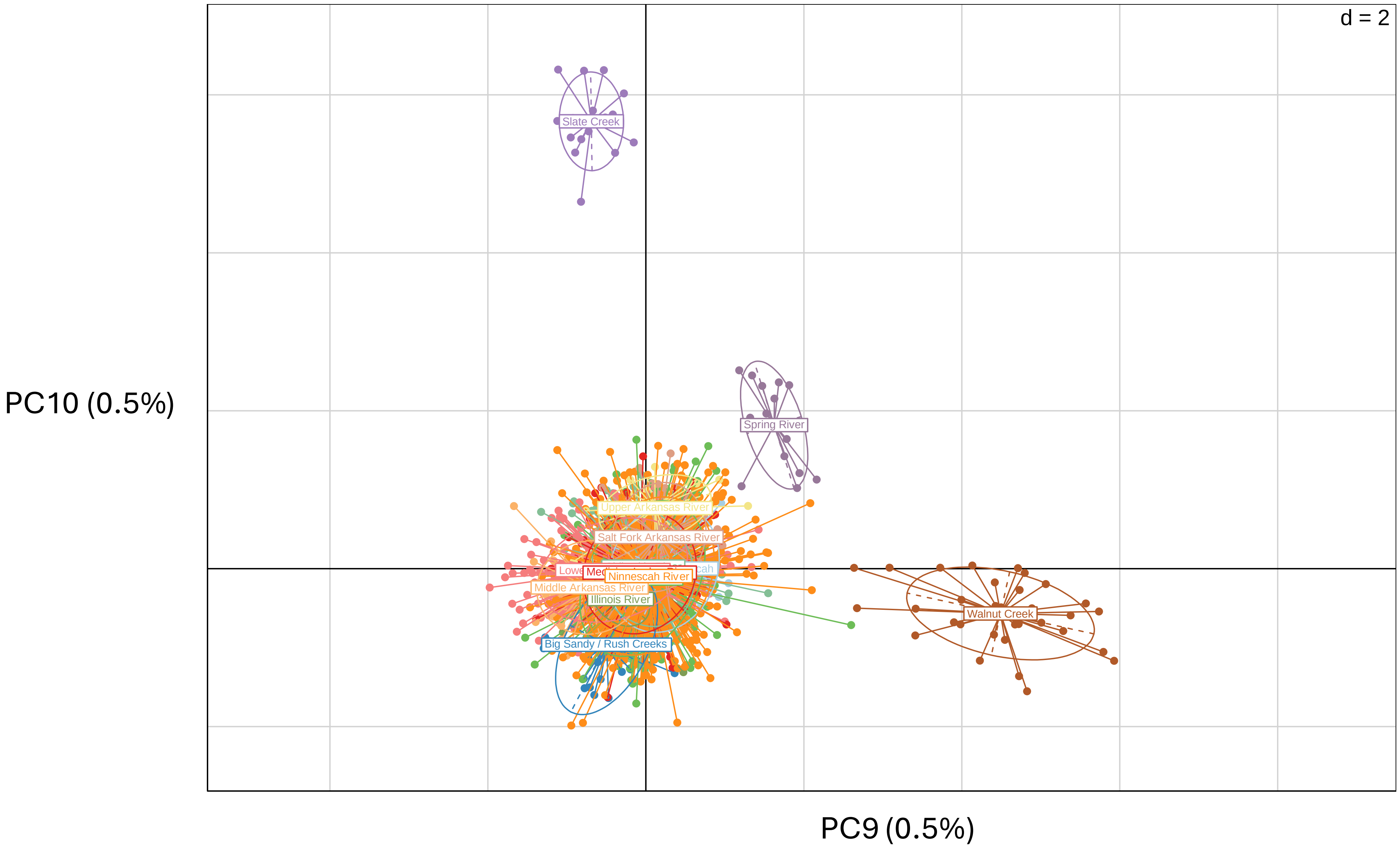

Supplemental Figure 3f. Principal components analysis (PC11+ PC12)

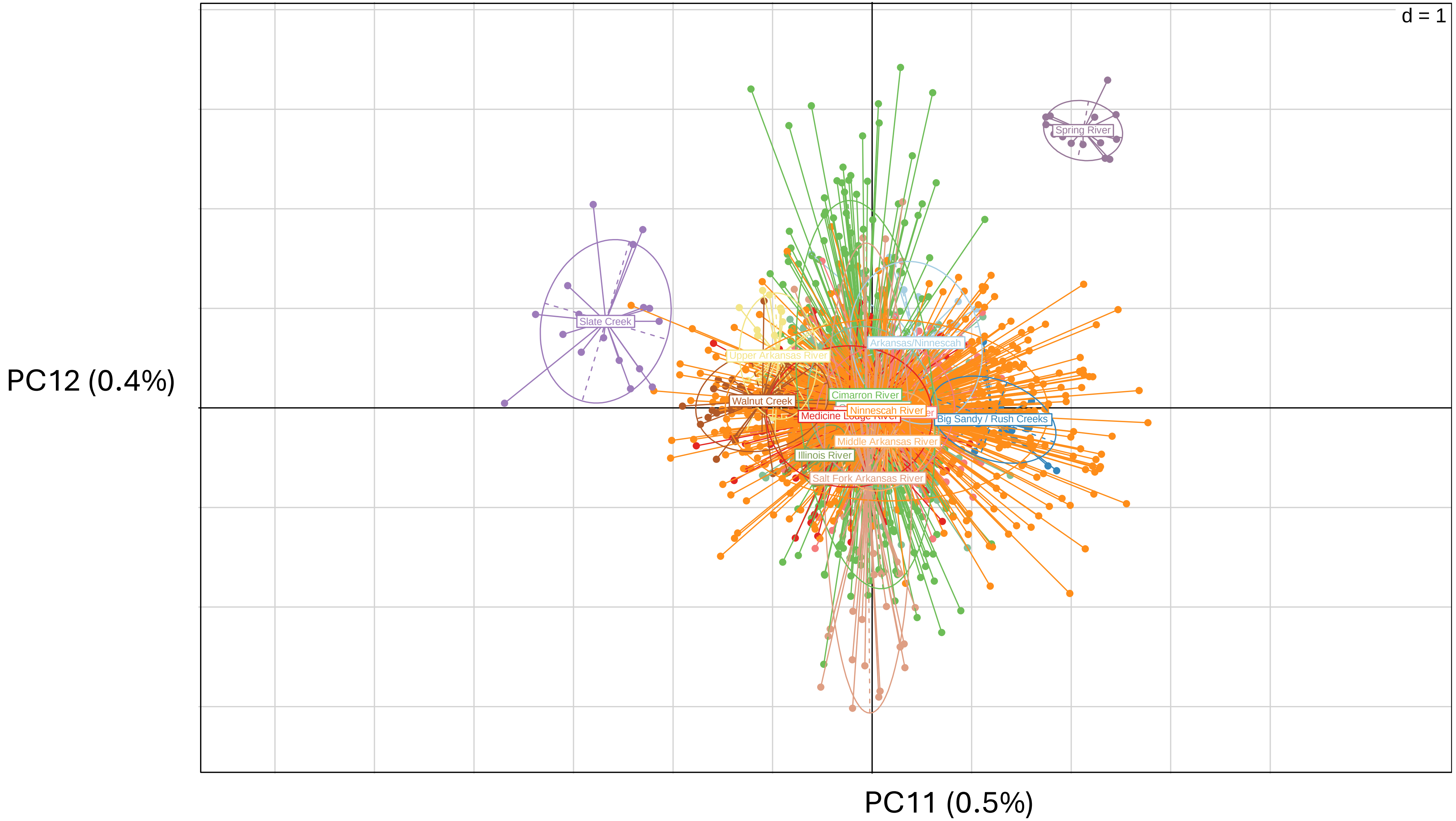

Supplemental Figure 3g. Principal components analysis (PC13+ PC14)

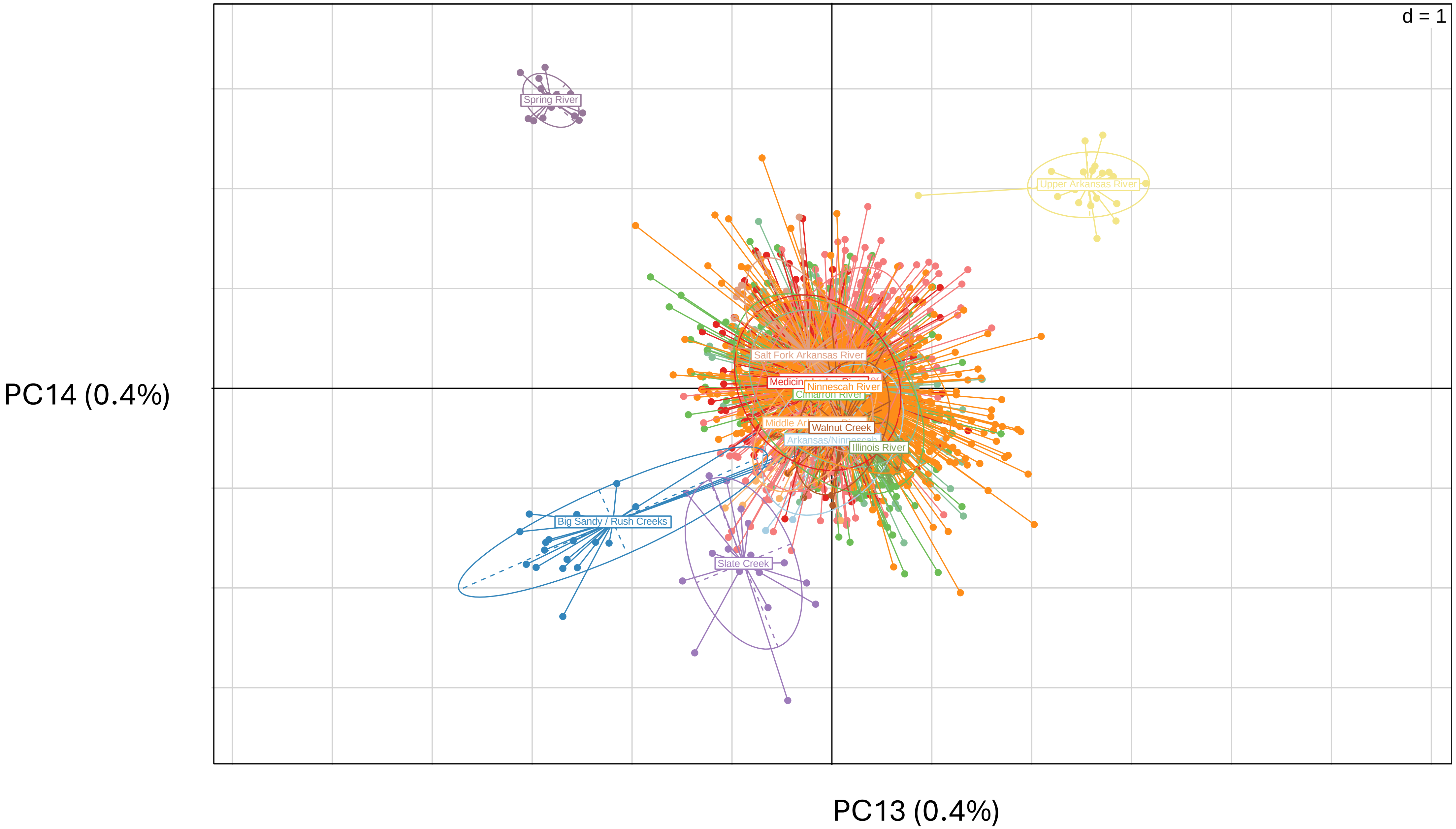

Supplemental Figure 3h. Principal components analysis (PC15+ PC16)

PC16 (0.3%)

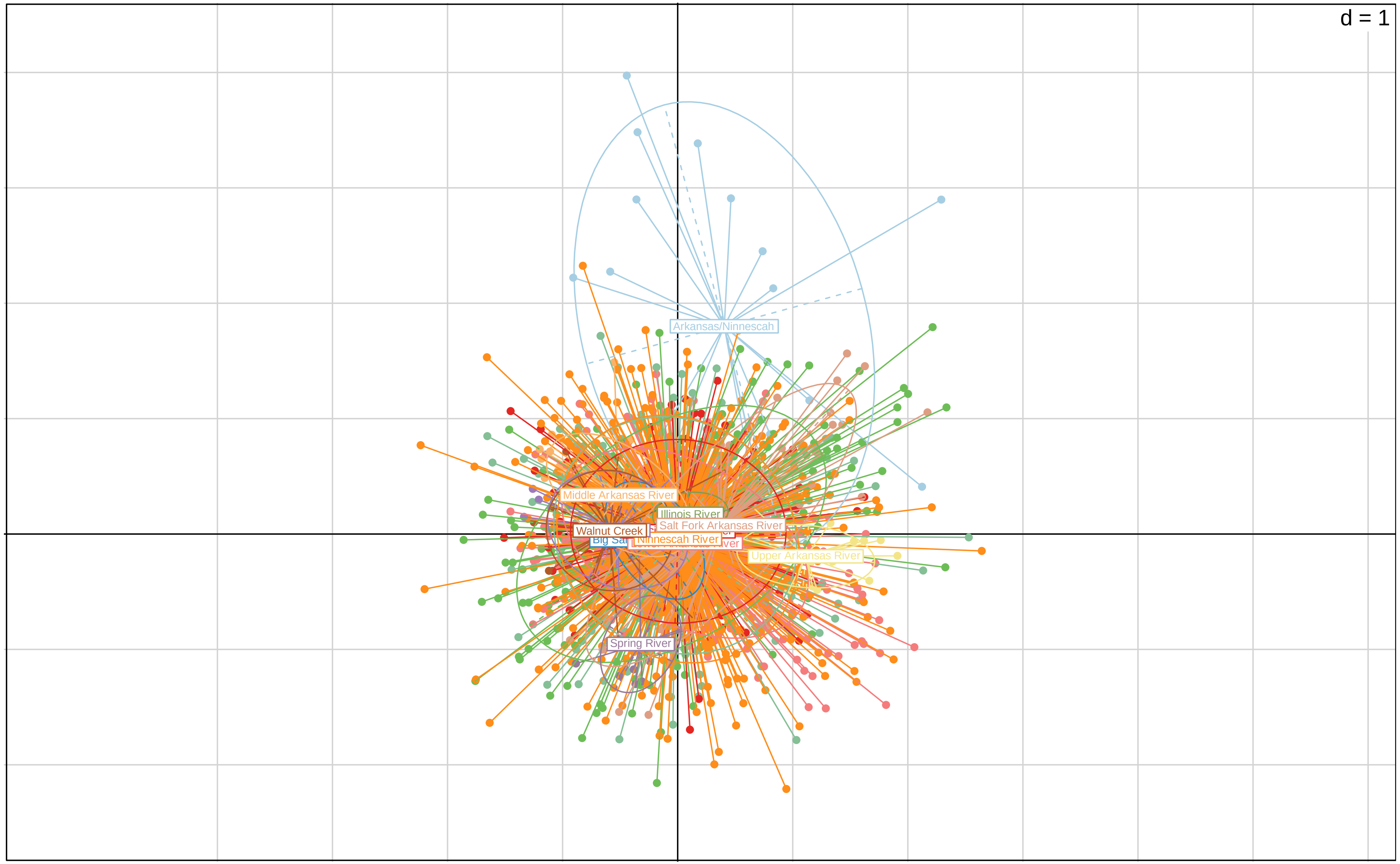

PC15 (0.3%)

Supplemental Figure 3i. Principal components analysis (PC17+ PC18)

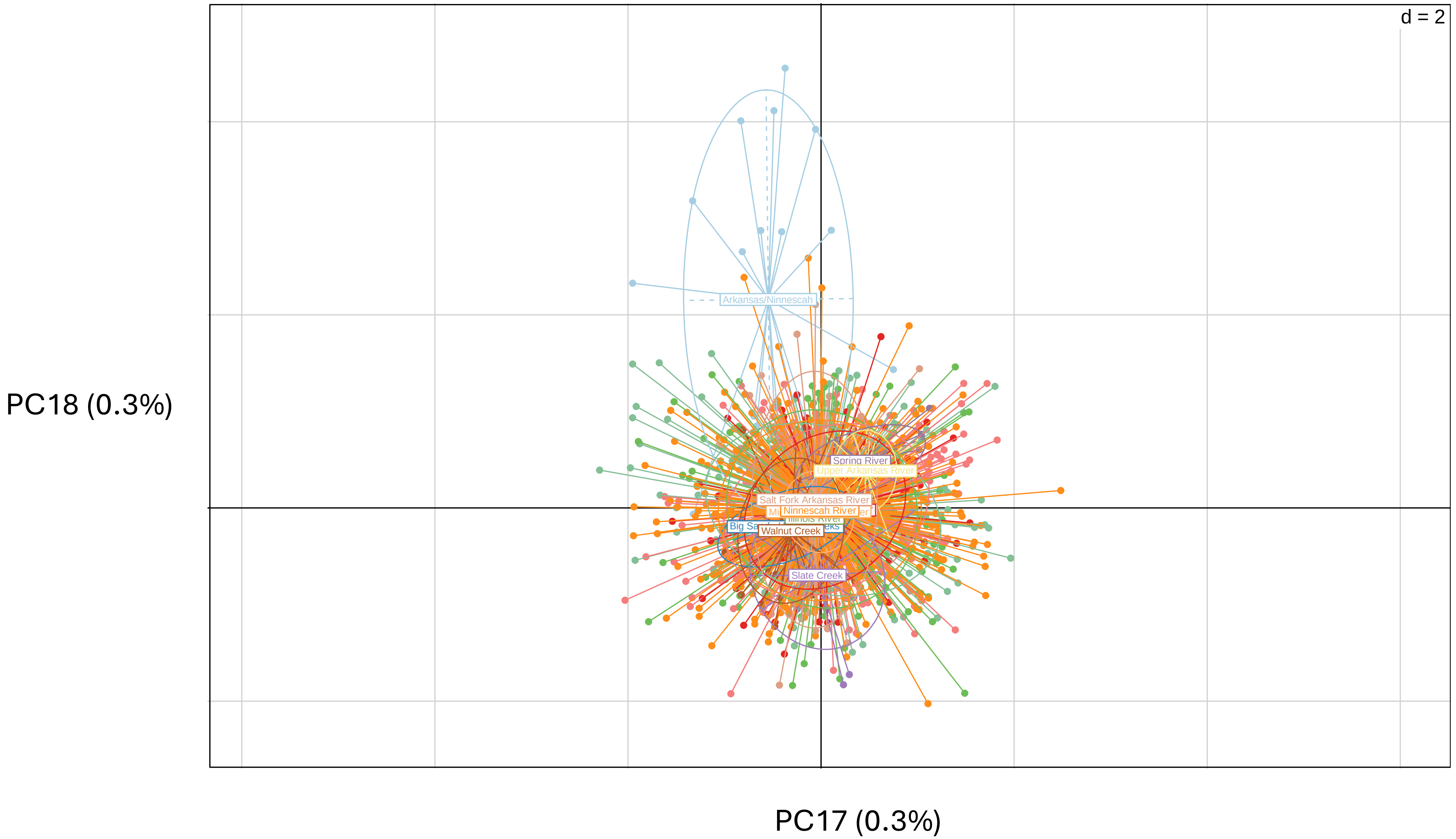

Supplemental Figure 3j. Principal components analysis (PC19+ PC20)

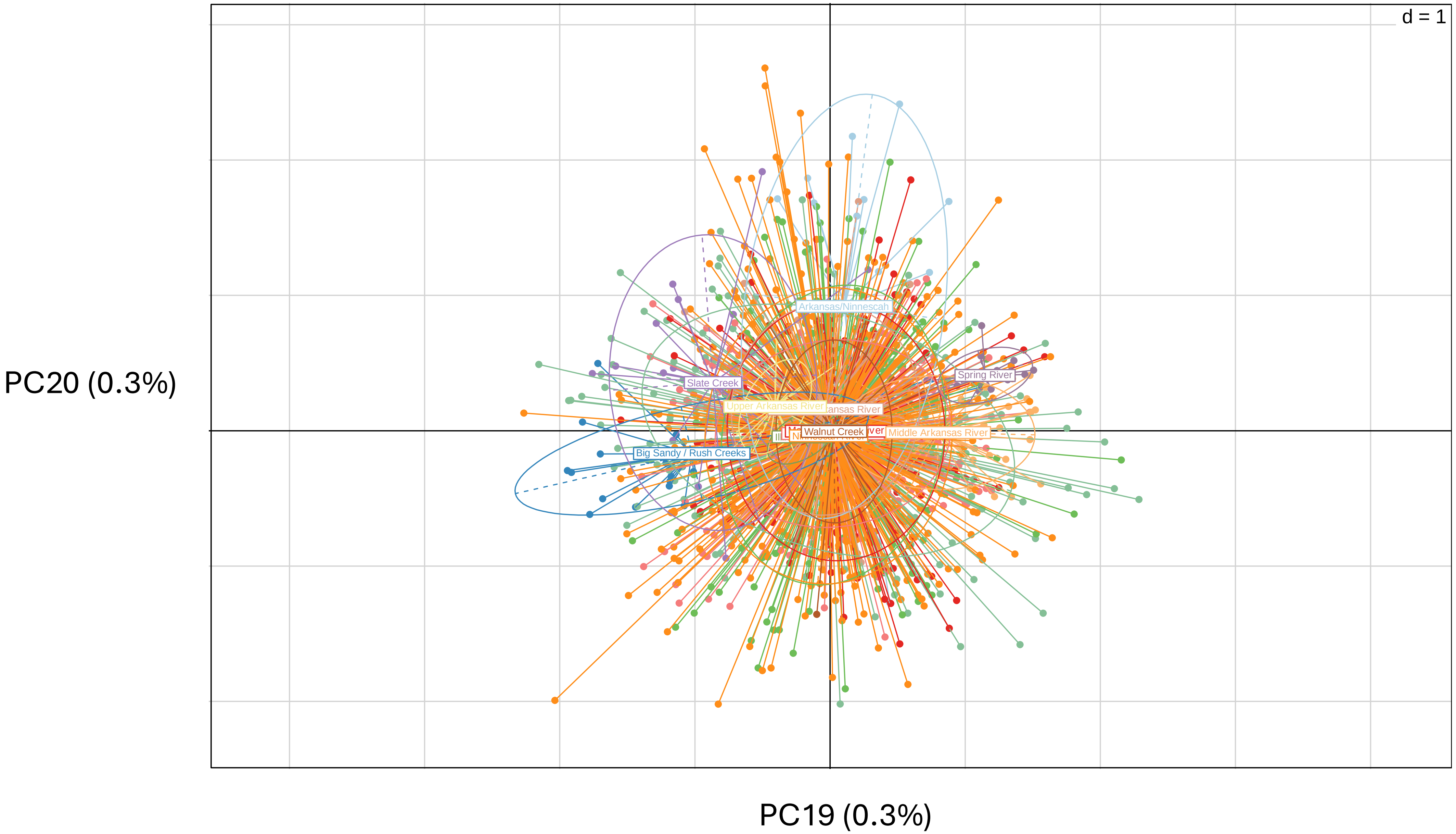

Supplement: Supplementary file 3 — Figure S3. PCA results showing the first 20 principal component axes for the Rapture dataset. Each genetically defined population is shown in a distinct color, with individuals shown as dots and confidence ellipses. Percent variation explained is shown on each principal component axis. [file EVA-18-e70088-s002.pdf]
